# Uncommon Capnosane Diterpenes with Neuroprotective Potential from South China Sea Soft Coral *Sarcophyton boettgeri*

**DOI:** 10.3390/md20100602

**Published:** 2022-09-25

**Authors:** Ye-Qing Du, Jing Chen, Meng-Jun Wu, Hai-Yan Zhang, Lin-Fu Liang, Yue-Wei Guo

**Affiliations:** 1 School of Chinese Materia Medica, Nanjing University of Chinese Medicine, Nanjing 210023, China; 2 State Key Laboratory of Drug Research, Shanghai Institute of Materia Medica, Chinese Academy of Sciences, 555, Zu Chong Zhi Road, Zhangjiang Hi-Tech Park, Shanghai 201203, China; 3 College of Materials Science and Engineering, Central South University of Forestry and Technology, 498 South Shaoshan Road, Changsha 410004, China; 4 Shandong Laboratory of Yantai Drug Discovery, Bohai Rim Advanced Research Institute for Drug Discovery, Yantai 264117, China; 5 Open Studio for Druggability Research of Marine Natural Products, Pilot National Laboratory for Marine Science and Technology (Qingdao), 1 Wenhai Road, Aoshanwei, Jimo, Qingdao 266237, China; 6 Collaborative Innovation Center of Yangtze River Delta Region Green Pharmaceuticals and College of Pharmaceutical Science, Zhejiang University of Technology, Hangzhou 310014, China

**Keywords:** soft coral, *Sarcophyton boettgeri*, capnosane, absolute configuration, neuroprotective effect

## Abstract

The first investigation of the South China Sea soft coral *Sarcophyton boettgeri* afforded five new capnosane diterpenes, sarboettgerins A–E (**1**–**5**), together with one known related compound, pavidolide D (**6**). Their structures, including absolute configurations, were elucidated by the extensive spectroscopic analysis, ^13^C NMR calculations, and X-ray diffraction. Among them, new compounds **1**–**5** were featured by the rarely encountered *Z*-geometry double bond Δ^1^ within the 5/11-fused bicyclic capnosane carbon framework. Plausible biogenetic relationships of all isolates were proposed, and they might give an insight into future biomimetic synthesis of these novel compounds. In an in vitro bioassay, compound **5** displayed potent anti-neuroinflammatory activity against LPS-induced NO release in BV-2 microglial cells, which might be developed as a new type of potential neuroprotective agent in future.

## 1. Introduction

Cembranoids are a vast family of natural diterpenes mainly found in terrestrial (e.g., plants of the genera *Nicotiana* [1] and *Pinus* [2]) and marine (e.g., soft corals of the genera *Sarcophyton* [3,4], *Sinularia* [5], *Lobophytum* [6], and *Alcyonium* [7,8]) organisms. The head-to-tail cyclization of a geranylgeranyl diphosphate (GGPP) is considered to generate a 14-membered marcocyclic carbon framework named cembrane [9]. Initiated by the 1,14-cyclisation of a GGPP precursor, the following bond formations of cembrane lead to a range of polycyclic diterpenes with diverse scaffolds, including 1,2-cyclized casbanes [10], 2,11-cyclized eunicellins [11], 3,7-cyclized capnosanes [12,13], and 2,20-cyclized sarsolenanes [12,13], as well as the newly discovered carbocyclic skeletons [14,15]. Additionally, the bond cleavages of cembrane afford an array of norcembranoids [16,17] and seco-cembranes [18,19]. These derivatives might originate from various types of enzymatic and/or non-enzymatic reactions, such as the Diels–Alder reaction, Michael addition, Pinacol rearrangement, Baeyer–Villiger oxidation, etc. [16,17]. From a pharmaceutical point of view, cembranoids have been credited with a broad range of biological activities, including neuroprotective, ichthyotoxic, anticancer, antifouling, antimicrobial, and anti-inflammatory activities [1,3,4,6,8,9,20]. Moreover, from an ecological point of view, some representative cembranoids are regarded as chemical defense tools to protect soft corals against natural predators, servicing as feeding deterrents or acting by virtue of their toxicities [21]. Because cembranoids exhibit a huge potential for the development of various drug candidates with remarkable health and ecological benefits, numerous efforts toward the chemical modification and total synthesis of them were made [16,22,23,24].

The capnosane diterpenes, characterized by a 5/11-fused bicyclic carbon framework, are regarded as biogenetically derived from cembranoids by transannular cyclization or ring rearrangement [17,25]. Although cembranoids are frequently encountered in soft corals, capnosanes rarely occur. Therefore, only a few investigations of capnosane diterpenes have been reported and merely found in 13 species of soft corals till now, including *Sarcophyton trocheliophorum* [12,26,27], *S. solidum* [28], *S. elegans* [29], *S. mililatensis* [13], *Sinularia pavida* [30], *S. humilis* [31], *S. rigida* [32], *S. polydactyla* [33], *Lobophytum pauciflorum* [34], *Lobophytum* sp. [35], *Klyxum flaccidum* [36], *Cespitularia* sp. [37], and *Alcyonium coralloides* [38]. Interestingly, two cembrane-capnosane heterodimers were discovered in the soft coral *S. subviride* recently [39]. Their structural diversity is mainly attributed to the different degrees of unsaturation, derived by the hydroxyl, epoxide, ketone, lactone, olefinic bond, and other functionalities [27]. Among them, the *Z*-geometry double-bond Δ^1^ is uncommon, with very few representatives [26,27,32].

**Figure 1:**
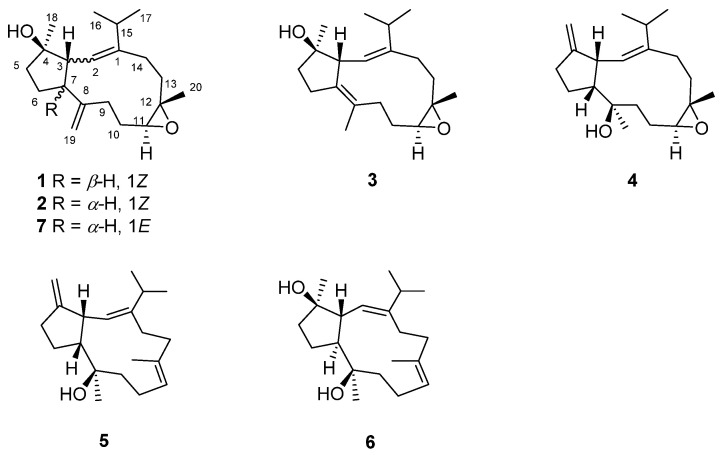
Structures of compounds **1**–**7**.

Soft corals of the genus *Sarcophyton* are well-known as a reservoir of diverse terpenes, including capnosanes [3]. As part of our ongoing project searching for bioactive secondary metabolites from Chinese marine cnidarians [40,41] and their predators [42], a recent investigation on the chemical constituents of *Sarcophyton boettgeri* collected from Weizhou Island, South China Sea, has afforded five new capnosane-type diterpenes, sarboettgerins A–E (**1**–**5**), along with one related known analog (**6**) (Figure 1). A survey of the literature revealed that there is no report regarding the chemical constituents of this species before this study. Herein, we describe the isolation, structural elucidation, bioactivity evaluation, and plausible biosynthetic pathway of the new compounds.

## 2. Results and Discussion

The frozen bodies of title animals were cut into pieces and extracted exhaustively with acetone. The Et_2_O-soluble portion of the acetone extract was repeatedly column chromatographed over silica gel, Sephadex LH-20, and RP-HPLC to yield five new compounds, i.e., compounds **1**–**5**, along with one known related compound, compound **6** (Figure 1). The known compound **6** was readily identified as pavidolide D, a capnosane diterpene isolated from the soft coral *S. pavida*, by comparison of the NMR data and optical rotation with those reported in the literature [30].

**Figure 2:**
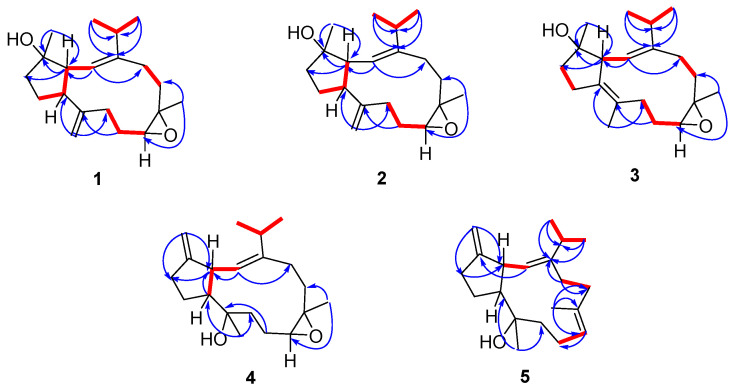
The selected key ^1^H–^1^H COSY (red lines) and HMBC (blue arrows, from ^1^H to ^13^C) correlations of compounds **1**–**5**.

**Figure 3:**
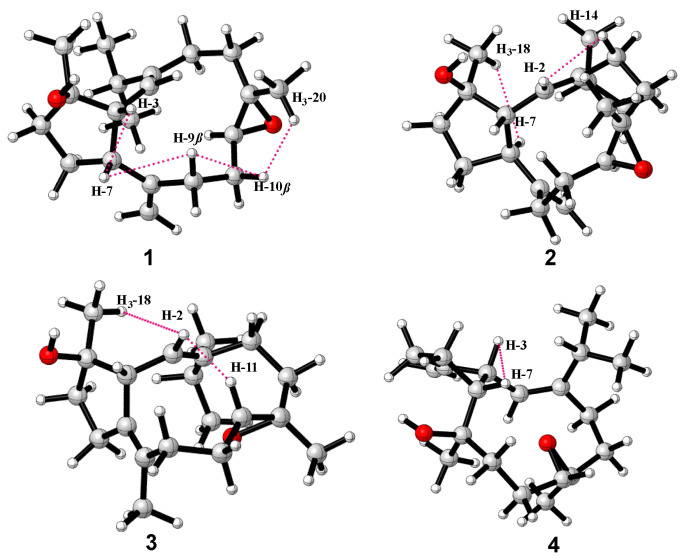
The selected key NOESY (red dashed lines, from ^1^H to ^1^H) correlations of compounds **1**–**4**.

**Figure 4:**
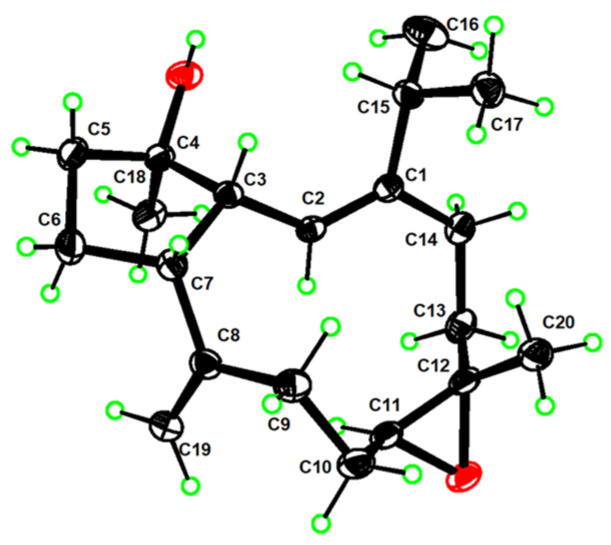
Perspective ORTEP drawing of X-ray structure of **1** (displacement ellipsoids are drawn at the 50% probability level).

Compound **1** was obtained as a colorless crystal and had the molecular formula C_20_H_32_O_2_ on the basis of its quasi-molecular ion peak at *m*/*z* 327.2299 ([M + Na]^+^, calcd. for C_20_H_32_O_2_Na, 327.2295), requiring four degrees of unsaturation. The IR absorption bands at 3452 and 1640 cm^−1^ revealed the existence of the hydroxyl and olefinic groups, which were in agreement with the presence of a tertiary alcohol functionality [*δ*_C_ 82.0 (qC, C-4)] and a terminal double bond [*δ*_H_ 4.92 (1H, s, H-19a) and 4.96 (1H, s, H-19b), *δ*_C_ 108.6 (CH_2_, C-19) and 149.1 (qC, C-8)], as indicated by the ^1^H and ^13^C NMR data (Table 1). Its ^13^C NMR spectrum exhibited twenty carbon resonances assigned to four methyls (*δ*_C_ 21.3, 20.8, 24.6, and 15.4), seven methylenes (including one olefinic, *δ*_C_ 108.6, and six aliphatic, *δ*_C_ 40.6, 26.7, 32.5, 24.9, 33.8, and 25.3), five methines (including one olefinic, *δ*_C_ 120.9; an oxygenated, *δ*_C_ 67.0; and three aliphatic, *δ*_C_ 28.9, 50.3, and 47.0), and four quaternary carbons (including two olefinic, *δ*_C_ 143.6 and 149.1; and two oxygenated, *δ*_C_ 82.0 and 59.3) (Table 1), which were deduced from DEPT and HSQC experiments. In addition, its ^1^H NMR spectrum displayed signals of an epoxide moiety at *δ*_H_ 3.15 (1H, dd, *J* = 10.7, 3.4 Hz, H-11), which corresponded to the ^13^C chemical shifts at *δ*_C_ 67.0 (CH, C-11) and 59.3 (qC, C-12) (Table 1). As revealed by the ^1^H and ^13^C NMR data, there were one double bond and one epoxide, accounting for two degrees of unsaturation. The remaining two degrees of unsaturation were due to the presence of two rings in the molecule. Considering the co-isolated secondary metabolite pavidolide D, compound **1** was likely a capnosane-type diterpene. Through a detailed literature review on diterpenes from soft corals, the abovementioned structural features were reminiscent of previously reported lobophytrol C (**7**) [35], a capnosane from South China Sea soft coral *Lobophytum* sp. Our interpretation of the 2D NMR spectra (Figure 2) suggested they shared the same gross structure. Indeed, the major differences were observed around C-1 (*δ*_C_ 143.6 for **1** vs. *δ*_C_ 148.1 for **7**), C-2 (*δ*_C_ 120.9 for **1** vs. *δ*_C_ 122.7 for **7**), and its neighboring carbons, C-3 (*δ*_C_ 50.3 for **1** vs. *δ*_C_ 51.8 for **7**), and C-7 (*δ*_C_ 47.0 for **1** vs. *δ*_C_ 54.4 for **7**), disclosing they were a pair of stereoisomers. The relative configuration of **1** was deduced from the analysis of its NOESY spectrum. As shown in Figure 3, the absence of NOE correlation between H-2 and H-15 indicated that the double-bond Δ^1^ in **1** took *Z*-geometry. Additionally, the key NOE cross-peaks of H-3/H-7, H-7/H-9*β*, H-9*β*/H-10*β*, and H-10*β/*H_3_-20, along with the lack of NOE interactions between H-3 and H_3_-18 and between H-11 and H_3_-20, revealed that H-3, H-7, and H_3_-20 were oriented on the same side of the ring system and tentatively assigned as *β*, while H-7, H-11, and H_3_-18 were *α*-oriented. In order to establish the absolute configuration of **1**, a suitable single crystal was obtained from methanol after many attempts. The successful X-ray diffraction experiments with Cu Ka (*λ*= 1.54178 Å) radiation allowed us to confirm the proposed structure and to determine its absolute configuration as 3*R*,4*R*,7*R*,11*S*,12*S* [Flack parameter: -0.06(7)] (Figure 4). Hereto, the structure determination of **1** was completely accomplished.

The HRESIMS spectrum of compound **2** displayed a quasi-molecular ion peak at *m/z* 303.2328 ([M–H]^–^, calcd. for C_20_H_31_O_2_, 303.2330), suggesting that **2** possessed the same molecular formula C_20_H_32_O_2_ as **1**. In fact, the ^1^H and ^13^C NMR data (Table 1) of **2** closely resembled those of **1**, and the 2D NMR (^1^H–^1^H COSY, HSQC, and HMBC) analysis revealed that both **2** and **1** had the same gross structure (Figure 2). The main difference between **2** and **1** was the significantly upfield-shifted H-7 (*δ*_H_ 2.19, m) in **2** in comparison with that (*δ*_H_ 2.88, m) of **1**, in addition to the substantially downfield shifted C-7 (*δ*_C_ 55.4) in **2**, indicating that **2** was a C-7 epimer of **1**. This hypothesis was ascertained by the examination of its NOESY data (Figure 3). The characteristic NOE cross-peak of H_3_-18/H-7 without the observation of H_3_-18/H-3 or H-7/H-3 revealed that H_3_-18 and H-7 were co-facial, while the orientations of H-3 and H-7 were *trans*. To further distinguish the epimer relationship between compounds **1** and **2**, quantum NMR calculations using DP4+ probability analysis were performed, which was a powerful tool for the structural determinations of natural products [43,44,45]. As the stereochemistry of **1** was corroborated by X-ray diffraction, the correctness of the prediction for **1** from using the DP4+ method was checked at first. Because the main concern of the stereochemistry was the *cis* orientation for H-3 and H-7, (3R*,4R*,7R*,11S*,12S*)-**1a** and (3R*,4R*,7S*,11S*, 12S*)-**1b** were selected as two initiative possible conformers and then subjected to quantum NMR calculations. As a result, the experimental NMR data of compound **1** best matched those of **1a** with 100% probability (Supplementary Figure S46), which agreed with that derived from X-ray diffraction. This result perfectly confirmed the reliability of the application of this method for capnosane-type diterpenes. The subsequent calculations of two possible isomers, (3R*,4R*,7R*,11S*,12S*)-**2a** and (3R*,4R*,7S*,11S*,12S*)-**2b**, were conducted. The result (Supplementary Figure S47) indicated that the experimental NMR data of (3R*,4R*,7S*,11S*,12S*)-**2b** were most consistent with the experimental data with DP4+ probability 100%, which confirmed our conclusion. Based on the biogenetic consideration and their nearly identical NMR chemical shifts, the absolute configuration of **2** could be assigned as 3*R*,4*R*,7*S*,11*S*,12*S*.

Compound **3** was obtained as a colorless oil. Its molecular formula was established as C_20_H_32_O_2_ by HRESIMS from the quasi-molecular ion peak at *m/z* 327.2293 ([M + Na]^+^, calcd. for C_20_H_32_O_2_Na, 327.2295), which was the same as that of **1**. A detailed analysis of the NMR data (Table 1) revealed the considerable structural similarity between **3** and **1**. They only differed in the migration of the double bond, in which the terminal Δ^8(19)^ in **1** was replaced by the endo Δ^7^ in **3**. This was supported by the HMBC correlations between H_3_-19 and C-7, C-8, and C-9 (Figure 2). As shown in its NOESY spectrum (Figure 3), H-2 exhibited NOE correlations with H_3_-18 and H-11, indicating that these protons were all co-facial. Meanwhile, the absence of NOE interaction between H_3_-18 and H-3 clearly clarified that their orientations were opposite. The *Z-*geometry of the 1,2-double bond was assigned due to the lack of NOE cross-peak of H-2/H-15. The *trans* orientations for H-11 and H_3_-20 were deduced from the ^13^C NMR shift value of C-19 (*δ*_C_ 16.1 < 20 ppm), which was confirmed by the absence of correlation between H-11 and H_3_-20. In light of these observations, the full structure of compound **3** was unambiguously established, as depicted in Figure 1.

**Figure 5:**
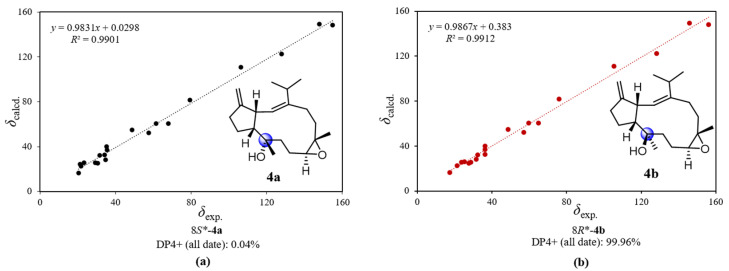
Regression analysis of experimental vs. calculated ^13^C NMR chemical shifts of (**a**) 8S*-**4a** and (**b**) 8R*-**4b** at the PCM/mPW1PW91/6-31+G** level, using DP4+ method.

The quasi-molecular ion peak at *m/z* 327.2291 ([M + Na]^+^, calcd. for C_20_H_32_O_2_Na, 327.2295) displayed in the HRESIMS spectrum revealed that compound **4** had the same molecular formula, C_20_H_32_O_2_, as that of the model compound **1**. A detailed analysis of NMR data (Table 1) of **4** suggested that **4** and **1** possessed the common moieties, including one trisubstituted double bond, one terminal bond, one tertiary alcohol functionality, and one epoxide group. Meanwhile, the extensive analyses of its ^1^H–^1^H COSY, HSQC, and HMBC spectra (Figure 2) disclosed that the positions of the terminal bond and the tertiary alcohol group were exchanged as compared to **1**. This was corroborated by the HMBC correlations between H_2_-18 and C-3 and C-5 and between H_3_-19 and C-7 and C-9. The geometry of double-bond Δ^1^, as well as its relative configurations at C-3, C-7, C-11, and C-12, was proven to be the same as that of **1** on the basis of a NOESY experiment (Figure 3). The absence of the NOE interaction from H-2 to H-15 clearly demonstrated the 1*Z* geometry. The NOESY correlations between H-3 and H-7 implied the *cis*-configuration of H-3 and H-7. The *trans* geometry of the epoxide group was confirmed by the absence of correlation between H-11 and H_3_-20. However, the NOESY experiment did not afford any useful correlations for the elucidation of the configuration of the chiral center C-8. To resolve this ambiguity, quantum NMR calculations of two possible isomers, 8S*-**4a** and 8R*-**4b**, using DP4+ probability analysis, were performed. As a result, the experimental NMR data of compound **4** gave the best match for **4b**, with 99.96% probability (Figure 5). Based on the above analysis, the structure of **4** was shown as depicted in Figure 1.

**Figure 6:**
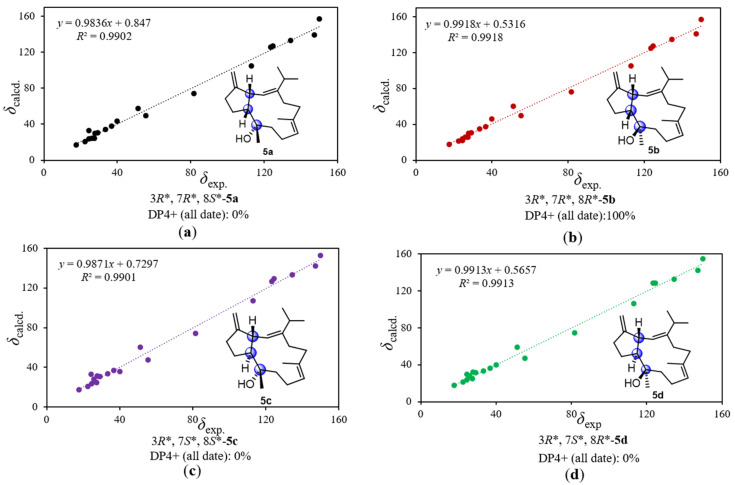
Regression analysis of experimental vs. calculated ^13^C NMR chemical shifts of (**a**) (3R*,7R*,8S*)-**5a**, (**b**) (3R*,7R*,8R*)-**5b**, (**c**) (3R*,7S*,8S*)-**5c**, and (**d**) (3R*,7S*,8R*)-**5d** at the PCM/mPW1PW91/6-31+G** level, using the DP4+ method.

Compound **5**, a colorless oil, had a molecular formula of C_20_H_32_O deduced by the molecular peak at *m/z* 288.2451 in the HREIMS spectrum ([M]^+^, calcd. for C_20_H_32_O, 288.2448), corresponding to five degrees of unsaturation. The NMR data of **5** were closely similar to those of **4**, except for the presence of one olefinic bond [*δ*_H_ 5.08 (1H, t, *J* = 10.5 Hz, H-11), *δ*_C_ 124.6 (CH, C-11) and 134.5 (qC, C-12)] in **5** instead of the epoxide group at C-11 and C-12 in **4**, as evidenced from their difference of 16 mass units. It was further validated by the HMBC correlations from H_3_-20 to C-11, C-13, and C-12 (Figure 2). As for the relative configuration of **5**, the absence of the NOE interaction between H-2 and H-15 clearly demonstrated the 1*Z* geometry (Figure 3). Meanwhile, the absence of the NOE cross-peak of H-11/H_3_-20 suggested the 11*E* geometry (Figure 3). Owing to the overlapped signals, the orientations of H-3, H-7, and H_3_-19 could not be determined via the NOESY experiment. Thus, quantum NMR calculations of the four possible conformers (3R*,7R*,8S*-**5a**, 3R*,7R*,8R*-**5b,** 3R*,7S*,8S*-**5c**, and 3R*,7S*,8R*-**5d**) were subjected to the GIAO method, at the mPW1PW91/6-31+G(d,p) level, with the PCM model in chloroform solvent (Figure 6). The result indicated that the experimental NMR data of (3R*,7R*,8R*)-**5b** were consistent with the experimental data, with a correlation coefficient *R*^2^ = 0.9918 and DP4+ probability of 100%. On the basis of the above analysis, compound **5** was depicted as shown in Figure 1.

**Scheme 1:**
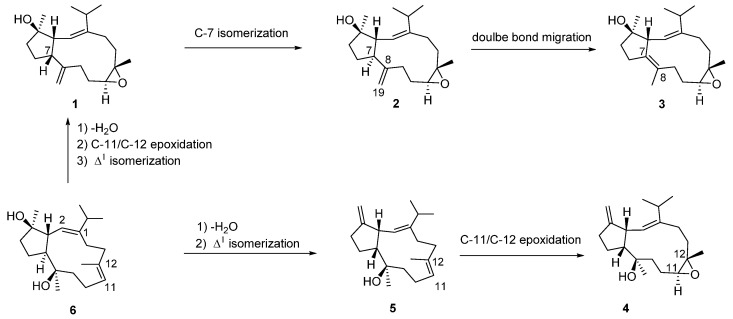
Proposed biosynthetic pathway of compounds **1**–**6**.

Compounds **1**–**6** shared the same 5/11-fused bicyclic carbon framework, namely capnosane. It is worth pointing out that the structural similarities between them and their co-occurrence suggested that they should originate via the same biogenetic pathway. Therefore, a plausible biosynthetic connection from the known compound **6** to all the new compounds was proposed based on their structural features (Scheme 1). The origin of **1** was proposed to be multiple possible biosynthetic pathways, including Δ^1^ isomerization, C-11/C-12 epoxidation, and a elimination of water from **6**. Compound **2** was thus derived from compound **1** by C-7 isomerization, which could undergo double-bond migration to afford compound **3**. Compound **5** was similarly generated by an elimination of water from **6**, followed by Δ^1^ isomerization. Subsequently, C-11/C-12 epoxidation on compound **5** yielded compound **4**.

Considering the interesting neuroprotective potential exhibited by some marine diterpenoids [3], the anti-neuroinflammatory activity of these isolates was assessed by employing lipopolysaccharide (LPS)-stimulated BV-2 microglial cells by measuring the release of nitric oxide (NO) levels in the supernatants. As shown in Table 2, compound **5** exhibited a statistically significant inhibitory effect on LPS-induced NO release in BV-2 microglial cells, and the level of NO decreased to 53.57 ± 1.24% at 20 μM and 13.30 ± 3.21% at 40 μM, respectively, comparable to that of the positive control, resveratrol (49.93 ± 6.66%), at 20 μM (NO data were normalized by the value of each LPS group). Compounds **3** and **4** (40 μM) displayed moderate anti-neuroinflammatory activities in LPS-stimulated BV-2 microglial cells when the level of NO decreased to 67.33 ± 4.11% and 39.03 ± 2.31%, respectively. In addition, all the isolates at 20 μM and 40 μM did not show marked cytotoxicity on BV-2 cells, suggesting that the anti-neuroinflammatory activity of the abovementioned active compounds was not attributed to their cytotoxicity. The excessive microglial inflammation induced by a variety of pathological insults could drive the progressive loss of neurons that occurs in multiple neurodegenerative diseases [46,47,48], and the abovementioned active isolates that potently suppress neuroinflammation are be expected to play a critical role in preventing and treating neuronal damages and considered to have neuroprotective potential.

## 3. Materials and Methods

### 3.1. General Experimental Procedures

The IR spectrum was recorded on a Nicolet iS50 spectrometer (Thermo Fisher Scientific, Madison, WI, USA). Optical rotations were measured on a PerkinElmer 241MC polarimeter. ^1^H and ^13^C NMR spectra were acquired on a Bruker AVANCE III 600 MHz spectrometer. Chemical shifts are reported with the residual CHCl_3_ (*δ*_H_ 7.26 ppm; *δ*_C_ 77.16 ppm) as the internal standard for ^1^H and ^13^C NMR spectra. HRESIMS spectra were recorded on Agilent G6250 Q-TOF (Agilent, Santa Clara, CA, USA). Commercial silica gel (Qingdao Haiyang Chemical Co., Ltd., Qingdao, China, 200–300 mesh, 300–400 mesh) was used for column chromatography, and precoated silica gel GF254 plates (Sinopharm Chemical Reagent Co., Shanghai, China) were used for analytical TLC. Sephadex LH-20 (Amersham Biosciences, Piscataway, NJ, USA) was also used for column chromatography. Reverse-phase (RP) HPLC was performed on an Agilent 1260 series liquid chromatography equipped with a DAD G1315D detector at 210 nm (Agilent, Santa Clara, CA, USA). An Agilent semi-preparative XDB-C18 column (5 μm, 250 × 9.4 mm) was employed for the purification. All solvents used for column chromatography and HPLC were of analytical grade (Shanghai Chemical Reagents Co., Ltd., Shanghai, China) and chromatographic grade (Dikma Technologies Inc., Foothill Ranch, CA, USA), respectively.

### 3.2. Animal Material

The soft coral *Sarcophyton boettgeri* was collected off the coast of Weizhou Island, Hainan Province, China, in 2011, at a depth of −20 m. The animal material was identified by Prof. Xiu-Bao Li from Hainan University. A voucher specimen (No. 11WZ-34) is available for inspection at the Shanghai Institute of Materia Medica.

### 3.3. Extraction and Isolation

The frozen animals (200.5 g, dry weight) were cut into pieces and extracted exhaustively with acetone at room temperature (3 × 3.0 L, 20 min in ultrasonic bath). The extract was concentrated under reduced pressure to give extractum. The extractum was partitioned between Et_2_O (1 L) and water (0.5 L). The Et_2_O-soluble portion was concentrated in vacuo to give a dark brown residue (11.0 g), which was fractionated by silica gel column chromatography and eluted with a step gradient (0–100% diethyl ether (EE) in petroleum ether (PE)), yielding seven fractions (A–G). Fraction D (2.4 g) was initially fractioned by Sephadex LH-20 column chromatography eluting with PE/CH_2_Cl_2_/CH_3_OH (2:1:1) to give four sub-fractions. The purification of sub-fraction D4 by silica gel column chromatography eluting with PE/EE yielded three sub-fractions, of which D4-3 was further purified by RP-HPLC (CH_3_CN/H_2_O, 75:25, 2.5 mL/min) to give compounds **1** (2.7 mg; *t*_R_ = 11.0 min) and **3** (2.1 mg; *t*_R_ = 12.3 min). Purification of sub-fraction D4-2 by RP-HPLC (CH_3_CN/H_2_O, 70:30, 2.5 mL/min) to afford compound **2** (1.8 mg, *t*_R_ =15.5 min). The sub-fraction D4-1 was purified by semipreparative HPLC with CH_3_CN/H_2_O (75:25, *v*/*v*, 2.5 mL/min) to afford compound **6** (4.0 mg, *t*_R_ = 9.6 min). Fraction E (0.7 g) was initially fractioned by Sephadex LH-20 column chromatography eluting with PE/CH_2_Cl_2_/CH_3_OH (2:1:1) to give two sub-fractions. Purification of sub-fraction E2 by RP-HPLC (CH_3_CN/H_2_O, 80:20) yielded compounds **4** (1.2 mg, *t*_R_ = 8.7 min) and **5** (4.4 mg, *t*_R_ = 11.2 min).

### 3.4. Spectroscopic Data of Compounds

Sarboettgerin A (**1**): colorless crystal; ${\left[\alpha \right]}_{D}^{20}$ −25.0 (*c* 0.04, CHCl_3_); IR: *ν*_max_ 3451, 2958, 2930, 2869, 1640, 1456, 1379, 1208, 1142, 1072, 933, 889 cm^−1^; ^1^H and ^13^C NMR data see Table 1; HRESIMS *m/z* 327.2299 [M+Na]^+^ (calcd. for C_20_H_31_O, 327.2295).

Ssarboettgerin B (**2**): colorless oil; ${\left[\alpha \right]}_{D}^{20}$ +36.2 (*c* 0.08, CHCl_3_); IR: *ν*_max_ 3470, 2958, 2928, 2862, 1741, 1455, 1378, 1238 cm^−1^; ^1^H and ^13^C NMR data see Table 1; HRESIMS *m/z* 303.2328 [M−H]^−^ (calcd. for C_20_H_31_O, 303.2330).

Sarboettgerin *C* (**3**): colorless oil; ${\left[\alpha \right]}_{D}^{20}$ −83.33 (*c* 0.06, CHCl_3_); IR: *ν*_max_ 3439, 2958, 2929, 2867, 1455, 1382, 1356, 1081, 905, 846, 817 cm^−1^; ^1^H and ^13^C NMR data see Table 1; HRESIMS *m/z* 327.2293 [M+H]^+^ (calcd. for C_20_H_32_NaO_2_, 327.2295).

Sarboettgerin D (**4**): colorless oil; ${\left[\alpha \right]}_{D}^{20}$ −33.0 (*c* 0.2, CHCl_3_); IR: *ν*_max_ 3418, 2957, 2926, 2869, 1715, 1456, 1377, 1096 cm^−1^; ^1^H and ^13^C NMR data see Table 1; HRESIMS *m/z* 327.2291 [M+Na]^+^ (calcd. for C_20_H_32_NaO, 327.2295).

Sarboettgerin E (**5**): colorless oil; ${\left[\alpha \right]}_{D}^{20}$ −33.0 (*c* 0.2, CHCl_3_); IR: *ν*_max_ 3458, 2959, 2927, 2870, 1739, 1442, 1381, 1369, 1259, 1234, 1086, 1022, 967, 876, 800 cm^−1^; ^1^H and ^13^C NMR data see Table 1; HREIMS *m/z* 288.2451 [M]^+^ (calcd. for C_20_H_3__2_O, 288.2448).

### 3.5. Crystal Structure Analysis of 1

Block crystals of compound **1** were obtained from solvent methanol at room temperature. Single-crystal X-ray diffraction of **1** (0.18 × 0.11 × 0.05 mm^3^) was performed on a Bruker Apex II CCD diffractometer (Bruker AXS Inc., Karlsruhe, Germany), using Cu Kα radiation (*λ* = 1.54178 Å) at 170 K. The collected data integration and reduction were processed with SAINT software (V8.40A, 2016, Bruker AXS Inc., Karlsruhe, Germany), and multi-scan absorption corrections were performed by using the SADABS program (V2014/7, **2014**, Bruker AXS Inc., Karlsruhe, Germany). The structures were solved by direct methods, using SHELXTL (V2018/3, **2018**, George M. Sheldrick), and were refined on *F*^2^ by the full-matrix least-squares technique, using the SHELXL program package. The absolute configuration was determined on the basis of a Flack parameter of −0.06(7) (Supplementary Table S1). Crystallographic data for **1** in this article were deposited at the Cambridge Crystallographic Data Centre (deposition number CCDC 2203997). Copies of these data can be obtained free of charge via www.ccdc.cam.ac.uk/conts/retrieving.html (accessed on 29 August 2022) or from the Cambridge Crystallographic Data Centre (12 Union Road, Cambridge CB21EZ, UK. Fax: (+44) 1223-336-033. E-mail: deposit@ccdc.cam.ac.uk).

### 3.6. Computational Details

Conformational search was performed by using the torsional sampling (MCMM) approach and the OPLS_2005 force field with the conformational search in an energy window of 21 kJ/mol. Conformers above 1% Boltzmann populations were reoptimized at the B3LYP/6-311G(d,p) level with the IEFPCM solvent model for chloroform. A frequency analysis was also carried out to confirm that the reoptimized geometries were at the energy minima. Subsequently, NMR calculations were performed at the PCM/ mPW1PW91/6-31+G** level, as recommended for DP4+. NMR shielding constants were calculated by using the GIAO method. Finally, shielding constants were averaged over the Boltzmann distribution obtained for each stereoisomer and correlated with the experimental data.

### 3.7. Cell Cultures

BV-2 microglial cells were generously presented by Prof. Linyin Feng (Shanghai Institute of Materia Medica, Chinese Academy of Sciences, Shanghai, China). Cells were cultured in Dulbecco’s modified Eagle’s medium (Life Tech, Grand Island, NY, USA) supplemented with 10% heat-inactivated fetal bovine serum (Gibco, Grand Island, NY, USA), 100 U/mL penicillin, and 100 μg/mL streptomycin at 37 °C in a humidified atmosphere containing 5% CO_2_.

### 3.8. In Vitro Anti-Neuroinflammatory Activity Assay

BV-2 microglial cells were cultured in 96-well plates at a density of 2×10^5^ cells/well and incubated in a 37 °C incubator containing 5% CO_2_ for 24 h. BV-2 microglial cells were preincubated with 20 μM or 40 μM of test compounds for 2 h, followed by 100 ng/mL of LPS exposure for 24 h. Then the culture medium of each well (50 μL) was collected to react with the same volume of Griess buffer (Sigma-Aldrich, St. Louis, MO, USA) in a 96-well plate for 15 min, in the dark, at room temperature. The absorbance, at the wavelength of 540 nm, was detected with a microplate reader, and the level of nitrite was calculated according to a sodium nitrite standard curve. 

### 3.9. Cell Viability Determination

To measure the influence of isolates on cell viabilities, BV-2 microglial cells were seeded in 96-well plates at a density of 1.25×10^4^ cells/well and incubated in a 37 °C incubator containing 5% CO_2_ for 24 hours. BV-2 microglial cells were incubated with 20 μM and 40 μM of test compounds for 24 hours and then treated with 0.5 mg/mL MTT for 3 hours. After the removal of the culture medium, the cells were then incubated with 100% DMSO to dissolve formazan. The absorbance at the wavelength of 490 nm was detected with a microplate reader, and the cell viability was calculated.

## 4. Conclusions

The chemical investigation of the South China Sea soft coral *S. boettgeri* led to the discovery of six capnosane diterpenes **1**–**6**, of which five new ones furnished the uncommon *Z*-geometry olefinic bond, Δ^1^. Their structures, including the absolute stereochemistry, were elucidated through extensive spectroscopic analysis, quantum mechanical–NMR calculations with DP4+ probability analysis, and X-ray diffraction analysis. Although many cembranoids were reported from soft corals, capnosane-type diterpenes characterized by a 5/11-fused bicyclic carbon skeleton are infrequently encountered. To the best of our knowledge, this is the first report of chemical investigation on the soft coral *S. boettgeri*, which demonstrated that *S. boettgeri* was a potential source of structurally diverse capnosanes. In the bioassay, compound **5** displayed significant anti-neuroinflammatory activity against LPS-induced NO release in BV-2 microglial cells. Although the detailed mechanism of action is still undefined for the neuroprotective effect of compound **5**, this research identified this uncommon capnosane diterpene as new chemotype of potential neuroprotective agents against neuroinflammation in microglial cells, which might give an insight for the development of novel drug candidates.

## Figures and Tables

**Table 1:** ^1^H NMR (600 MHz) and ^13^C NMR (150 MHz) data for **1**–**5** in CDCl_3_ *.

No.	1		2		3		4		5	
	*δ*_H_ mult. (*J*, Hz)	*δ*_C_	*δ*_H_ mult. (*J*, Hz)	*δ*_C_	*δ*_H_ mult. (*J*, Hz)	*δ*_C_	*δ*_H_ mult. (*J*, Hz)	*δ*_C_	*δ*_H_ mult. (*J*, Hz)	*δ*_C_
1	-	143.6	-	143.2	-	139.8	-	149.2		147.3
2	4.92 d (10.8)	120.9	4.82 dd (9.6, 2.4)	124.8	4.81 d (9.6)	126.4	4.91 d (10.2)	122.5	4.88 d (10.2)	123.4
3	3.20 t (10.8)	50.3	2.80 dd (11.4, 9.6)	56.7	3.36 d (9.6)	52.4	2.43 m	54.7	2.39 overlap	55.4
4	-	82.0	-	80.8	-	82.6	-	148.2	-	149.9
5	1.82 m	40.6	1.94 m 1.75 m	40.8	1.70 m 1.63 m	39.2	1.67 m	25.9	1.82 m 2.17 m	27.8
6	1.87 m	26.7	1.56 overlap 1.96 overlap	32.2	2.48 m 2.37 m	29.2	2.57 t (13.2) 1.67 m	25.6	1.62 m	25.9
7	2.88 m	47.0	2.19 m	55.4	-	140.3	2.66 m	52.3	2.39 overlap	51.2
8	-	149.1		152.8	-	127.6	-	81.6	-	81.6
9	2.23 m 1.88 m	32.5	2.51 m 1.99 m	35.2	2.32 m 1.95 m	31.0	1.75 m	40.0	1.76 m 1.69 m	40.0
10	1.34 m 2.36 m	24.9	1.56 overlap 1.96 overlap	31.2	1.23 m 2.04 m	23.7	2.35 m 1.35 m	28.2	2.27 m 2.17 m	27.4
11	3.15 dd (10.8, 3.3)	67.0	2.89 dd (9.6, 1.5)	67.9	2.84 dd (11.4, 2.4)	66.5	2.88 dd (9.6, 4.5)	60.5	5.08 t (10.5)	124.6
12	-	59.3	-	61.0	-	59.9	-	60.6		134.5
13	1.48 m 2.12 m	33.8	2.11 m 1.56 overlap	34.9	1.48 td (13.5, 4.8) 2.11 m	35.3	2.06 m	37.2	2.06 m 1.79 m	36.8
14	2.21 m	25.3	2.23 m 2.05 m	23.8	2.04 m 2.24 m	23.8	1.94 m	24.7	2.43 m 1.94 m	29.2
15	3.10 m	28.9	2.95 m	29.8	3.03 m	29.6	2.15 m	32.7	2.20 m	33.4
16	1.01 d (6.9)	20.8	0.96 d (6.9)	20.6	1.05 d (6.9)	20.0	1.00 d (6.9)	24.5	1.03 d (6.9)	22.4
17	0.99 d (6.9)	21.3	0.98 d (6.9)	21.4	0.98 d (6.9)	20.5	1.02 d (6.6)	22.5	1.05 d (6.9)	24.5
18	1.09 s	24.6	1.14 s	26.3	1.23 s	24.9	4.96 s 4.89 s	110.1	4.86 s 4.79 s	113.0
19	5.20 s 5.11 s	108.6	4.92 s 4.96 s	110.8	1.67 s	18.3	1.12 s	25.0	1.08 s	24.4
20	1.14 s	15.4	1.09 s	16.3	1.18 s	16.1	1.38 s	16.6	1.62 s	17.6

* Assignments were deduced by analysis of 1D and 2D NMR spectra.

**Table 2:** The inhibitory effects of compounds **1**–**6** on LPS-induced NO production in BV-2 cells (20 and 40 μM).

Compounds	NO Production ^1^ (% of LPS Group) ± SEM	
	20 μM	40 μM
**1**	92.97 ± 5.44	81.07 ± 4.04 *
**2**	87.00 ± 3.16 *	73.73 ± 3.36 ***
**3**	82.63 ± 3.86 *	67.33 ± 4.11 ***
**4**	72.00 ± 0.81 ***	39.03 ± 2.31 ***
**5**	53.57 ± 1.24 ***	13.30 ± 3.21 ***
**6**	87.60 ± 2.55 **	71.00 ± 1.86 ***
Resveratrol (20 μM)	49.93 ± 6.66 **	-

^1^ NO data were normalized by the value of respective LPS group, which was set to 100%; * *p <* 0.05, ** *p <* 0.01, *** *p <* 0.001 vs. the respective LPS group; *n* = 3.

## Data Availability

Data are contained within the article or Supplementary Materials.

## Supplementary Materials

The following supporting information can be downloaded at https://www.mdpi.com/article/10.3390/md20100602/s1. Figures S1–S45: NMR, IR, and MS spectra of compounds **1**–**5**. Figures S46–S49: Regression analysis of experimental *vs* calculated ^13^C NMR chemical shifts of different conformers of compounds **1**, **2**, **6**, and **7** at the PCM/mPW1PW91/6-31+G** level using DP4+ method. Table S1: Crystal data and structure refinement for compound **1**.
