# Supplementary material for: Uncommon Capnosane Diterpenes with Neuroprotective Potential from South China Sea Soft Coral Sarcophyton boettgeri
Source: Mar Drugs. 2022 Sep 25;20(10):602. doi: 10.3390/md20100602 (PMC9604702; doi:10.3390/md20100602)
Supplement: Supplementary file 1 [file marinedrugs-20-00602-s001.zip › marinedrugs-1933722-supplementary.pdf]

Supporting Information

Uncommon Capnosane Diterpenes with Neuroprotective Potential from A New Collection of South China Sea Soft Coral *Sarcophyton boettgeri*

Ye-Qing Du[1,2,†], Jing Chen[1,2,†], Meng-Jun Wu[2,6], Hai-Yan Zhang[1,2,*], Lin-Fu Liang[3,*] and Yue-Wei Guo[1,2,4,5,6,*]

[1] School of Chinese Materia Medica, Nanjing University of Chinese Medicine, Nanjing 210023, China

[2] State Key Laboratory of Drug Research, Shanghai Institute of Materia Medica, Chinese Academy of Sciences, 555, Zu Chong Zhi Road, Zhangjiang Hi-Tech Park, Shanghai 201203, China

[3] College of Materials Science and Engineering, Central South University of Forestry and Technology, 498 South Shaoshan Road, Changsha 410004, China

[4] Shandong Laboratory of Yantai Drug Discovery, Bohai rim Advanced Research Institute for Drug Discovery, Yantai 264117, China

[5] Open Studio for Druggability Research of Marine Natural Products, Pilot National Laboratory for Marine Science and Technology (Qingdao), 1 Wenhai Road, Aoshanwei, Jimo, Qingdao 266237, China

[6] Collaborative Innovation Center of Yangtze River Delta Region Green Pharmaceuticals and College of Pharmaceutical Science, Zhejiang University of Technology, Hangzhou 310014, China

[*] Correspondence: hzhang@simm.ac.cn (H.-Y. Z.); lianglinfu@csuft.edu.cn (L.-F.L.); ywguo@simm.ac.cn (Y.-W.G.); Tel.: +86-21-50805813 (Y.-W.G.)

[†] These authors contributed equally to this work

Table of contents

Figure S1. ^{1}H NMR spectrum of **1** (600MHz, $CDCl_3$)
Figure S2. ^{13}C NMR spectrum of **1** (150 MHz, $CDCl_3$)
Figure S3. DEPT spectrum of **1** (150 MHz, $CDCl_3$)
Figure S4. ^{1}H–^{1}H COSY spectrum of **1** (600 MHz, $CDCl_3$)
Figure S5. HSQC spectrum of **1** (600 MHz, $CDCl_3$)
Figure S6. HMBC spectrum of **1** (600 MHz, $CDCl_3$)
Figure S7. NOESY spectrum of **1** (600 MHz, $CDCl_3$)
Figure S8. IR spectrum of **1**
Figure S9. HRESIMS spectrum of **1**
Figure S10. ^{1}H NMR spectrum of **2** (600MHz, $CDCl_3$)
Figure S11. ^{13}C NMR spectrum of **2** (150 MHz, $CDCl_3$)
Figure S12. DEPT spectrum of **2** (150 MHz, $CDCl_3$)
Figure S13. ^{1}H–^{1}H COSY spectrum of **2** (600 MHz, $CDCl_3$)
Figure S14. HSQC spectrum of **2** (600 MHz, $CDCl_3$)
Figure S15. HMBC spectrum of **2** (600 MHz, $CDCl_3$)
Figure S16. NOESY spectrum of **2** (600 MHz, $CDCl_3$)
Figure S17. IR spectrum of **2**
Figure S18. HRESIMS spectrum of **2**
Figure S19. ^{1}H NMR spectrum of **3** (600MHz, $CDCl_3$)
Figure S20. ^{13}C NMR spectrum of **3** (150 MHz, $CDCl_3$)
Figure S21. DEPT spectrum of **3** (150 MHz, $CDCl_3$)
Figure S22. ^{1}H–^{1}H COSY spectrum of **3** (600 MHz, $CDCl_3$)
Figure S23. HSQC spectrum of **3** (600MHz, $CDCl_3$)
Figure S24. HMBC spectrum of **3** (600 MHz, $CDCl_3$)
Figure S25. NOEY spectrum of **3** (600MHz, $CDCl_3$)
Figure S26. IR spectrum of **3**
Figure S27. HRESIMR spectrum of **3**
Figure S28. ^{1}H NMR spectrum of **4** (600MHz, $CDCl_3$)
Figure S29. ^{13}C NMR spectrum of **4** (150 MHz, $CDCl_3$)
Figure S30. DEPT spectrum of **4** (150 MHz, $CDCl_3$)
Figure S31. ^{1}H–^{1}H COSY spectrum of **4** (600 MHz, $CDCl_3$)
Figure S32. HSQC spectrum of **4** (600 MHz, $CDCl_3$)
Figure S33. HMBC spectrum of **4** (600 MHz, $CDCl_3$)
Figure S34. NOESY spectrum of **4** (600 MHz, $CDCl_3$)
Figure S35. IR spectrum of **4**
Figure S36. HRESIMR spectrum of **4**
Figure S37. ^{1}H NMR spectrum of **5** (600MHz, $CDCl_3$)
Figure S38. ^{13}C NMR spectrum of **5** (150 MHz, $CDCl_3$)
Figure S39. DEPT spectrum of **5** (150 MHz, $CDCl_3$)
Figure S40. ^{1}H–^{1}H COSY spectrum of **5** (600 MHz, $CDCl_3$)
Figure S41. HSQC spectrum of **5** (600 MHz, $CDCl_3$)
Figure S42. HMBC spectrum of **5** (600 MHz, $CDCl_3$)

Figure S43. NOESY spectrum of **5** (600 MHz, $CDCl_3$)
Figure S44. IR spectrum of **5**
Figure S45. HREIMR spectrum of **5**
Figure S46. Regression analysis of experimental *vs* calculated ^{13}C NMR chemical shifts of (3*R**,4*R**,7*R**,11*S**,12*S**)-**1a**, (3*R**,4*R**,7*S**,11*S**,12*S**)-**1b** at the PCM/mPW1PW91/6-31+G** level using DP4+ method
Figure S47. Regression analysis of experimental *vs* calculated ^{13}C NMR chemical shifts of (3*R**,4*R**,7*R**,11*S**,12*S**)-**2a**, (3*R**,4*R**,7*S**,11*S**,12*S**)-**2b** at the PCM/mPW1PW91/6-31+G** level using DP4+ method
Figure S48. Regression analysis of experimental *vs* calculated ^{13}C NMR chemical shifts of (3*R**,4*R**,7*R**,8*R**)-**6a**, (3*R**,4*R**,7*S**,8*R**)-**6b** at the PCM/mPW1PW91/6-31+G** level using DP4+ method
Figure S49. Regression analysis of experimental *vs* calculated ^{13}C NMR chemical shifts of (3*R**,4*R**,7*S**,11*S**,12*S**)-**7a**, (3*R**,4*R**,7*R**,11*S**,12*S**)-**7b** at the PCM/mPW1PW91/6-31+G** level using DP4+ method
Table S1 Crystal data and structure refinement for compound **1**

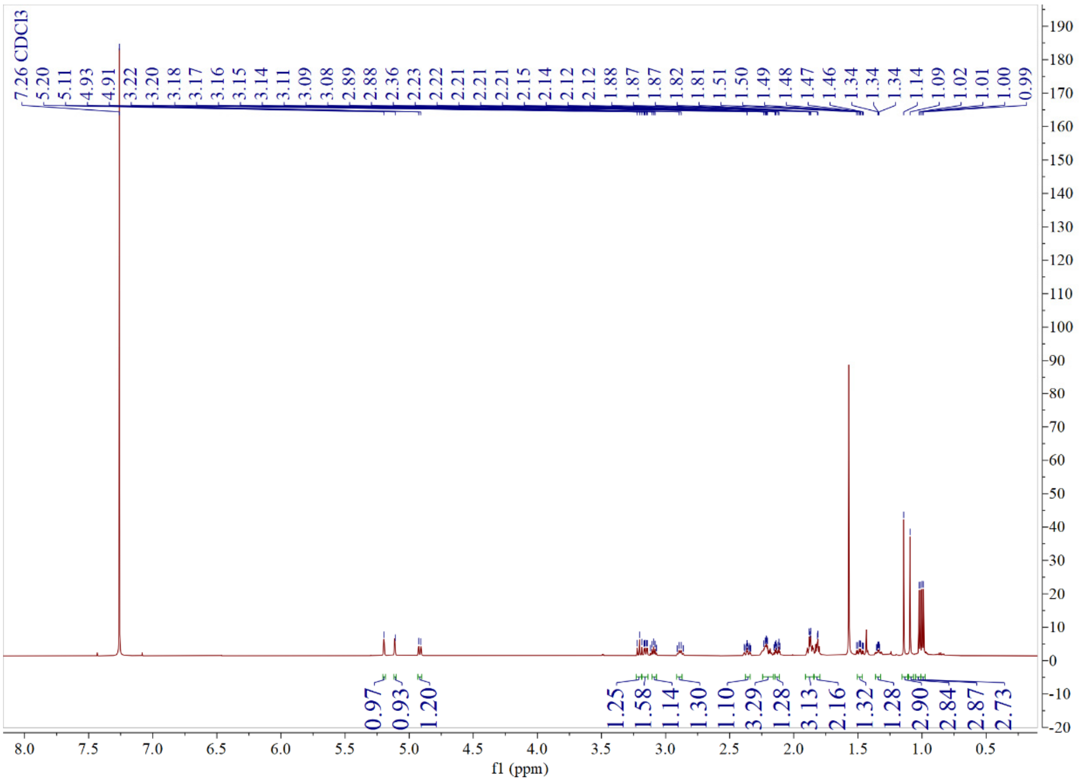

Figure S1. ^{1}H NMR spectrum of **1** (600MHz, $CDCl_3$)

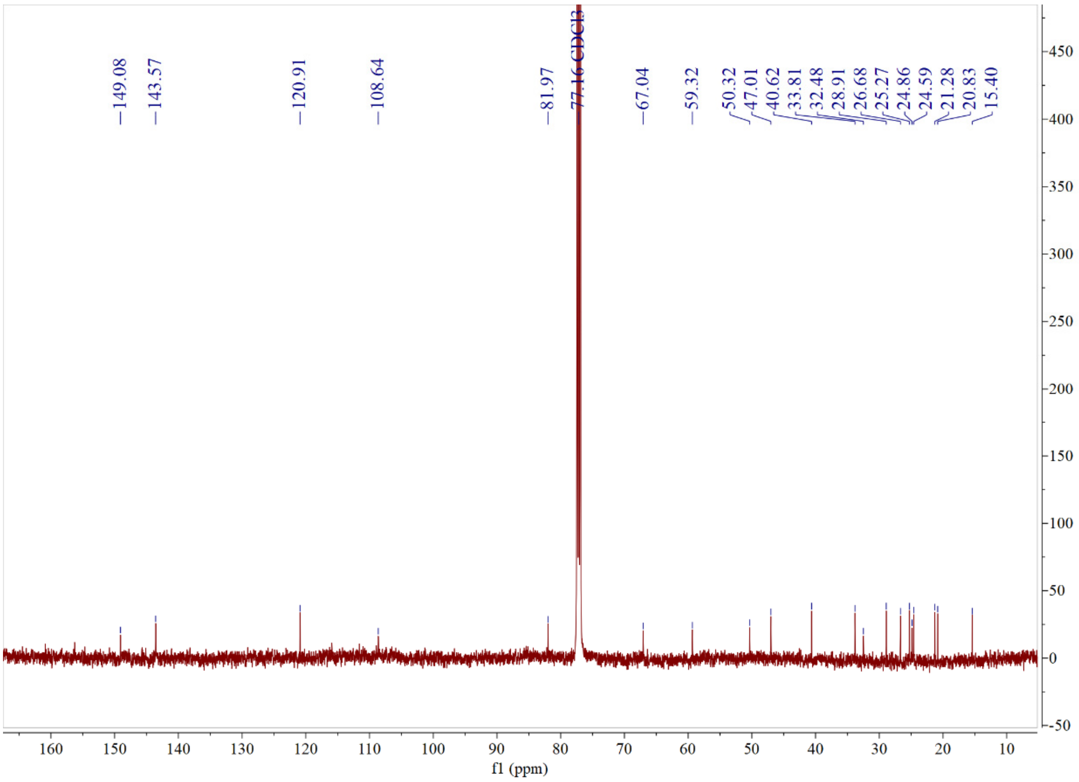

Figure S2. ^{13}C NMR spectrum of **1** (150 MHz, $CDCl_3$)

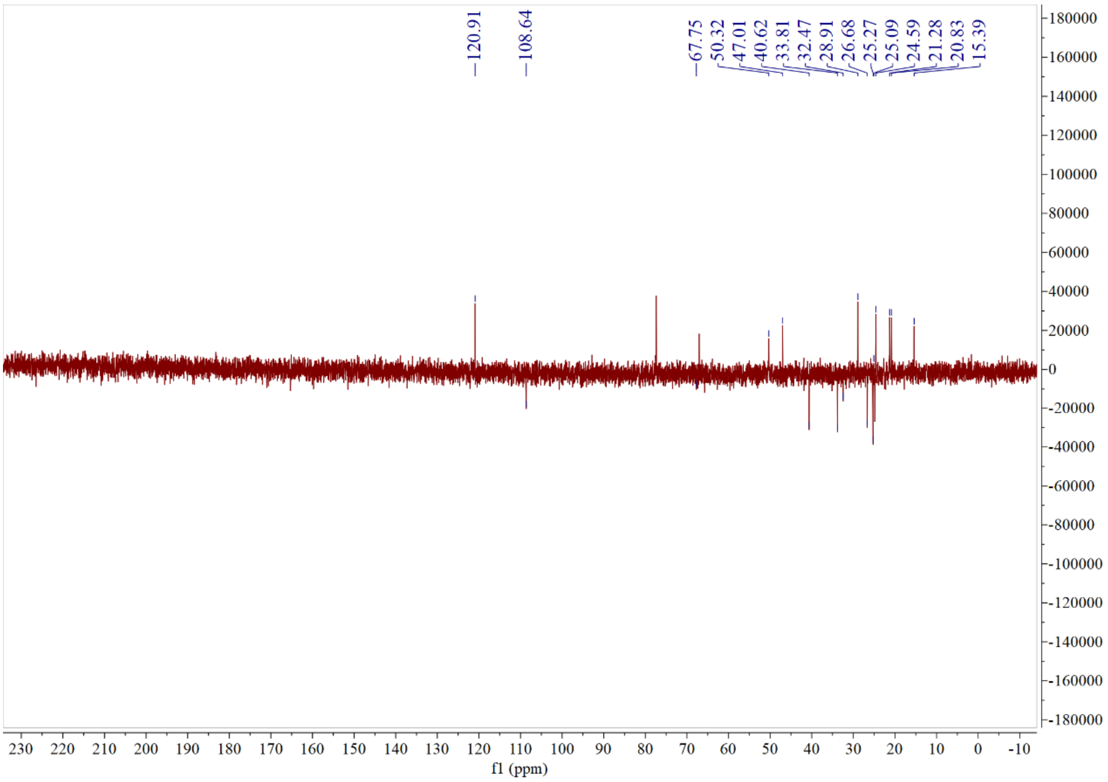

Figure S3. DEPT spectrum of **1** (150 MHz, $CDCl_3$)

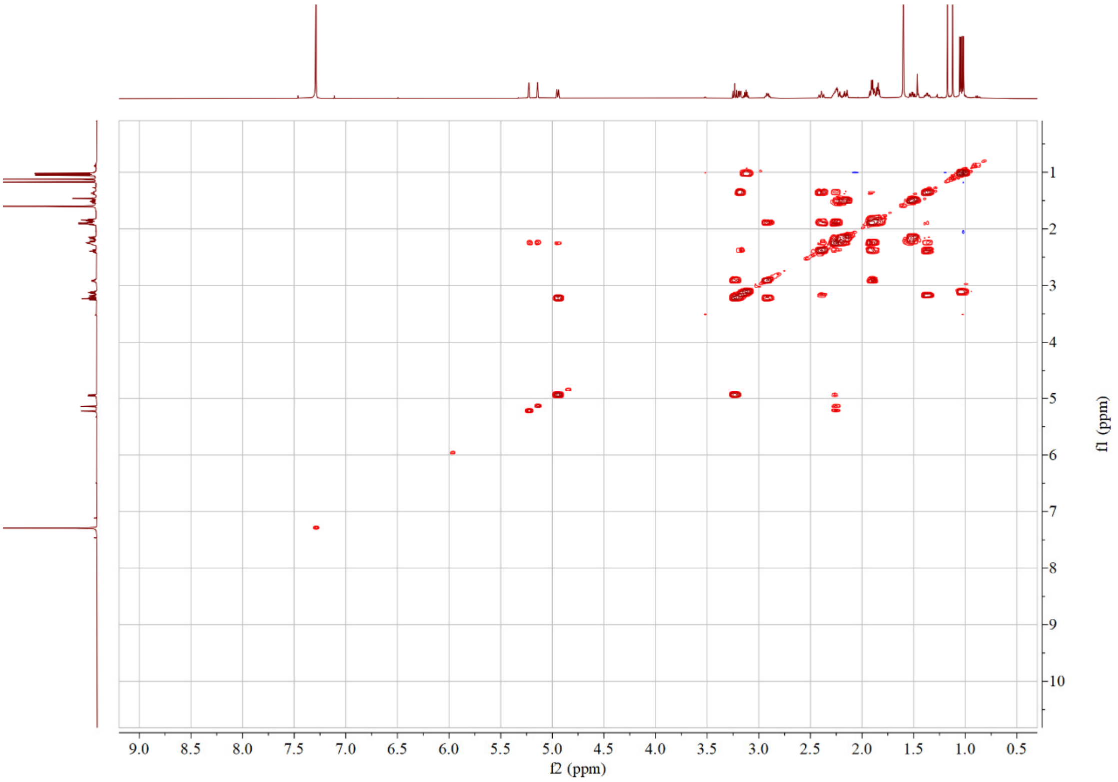

Figure S4. ^1H–^1H COSY spectrum of **1** (600 MHz, $CDCl_3$)

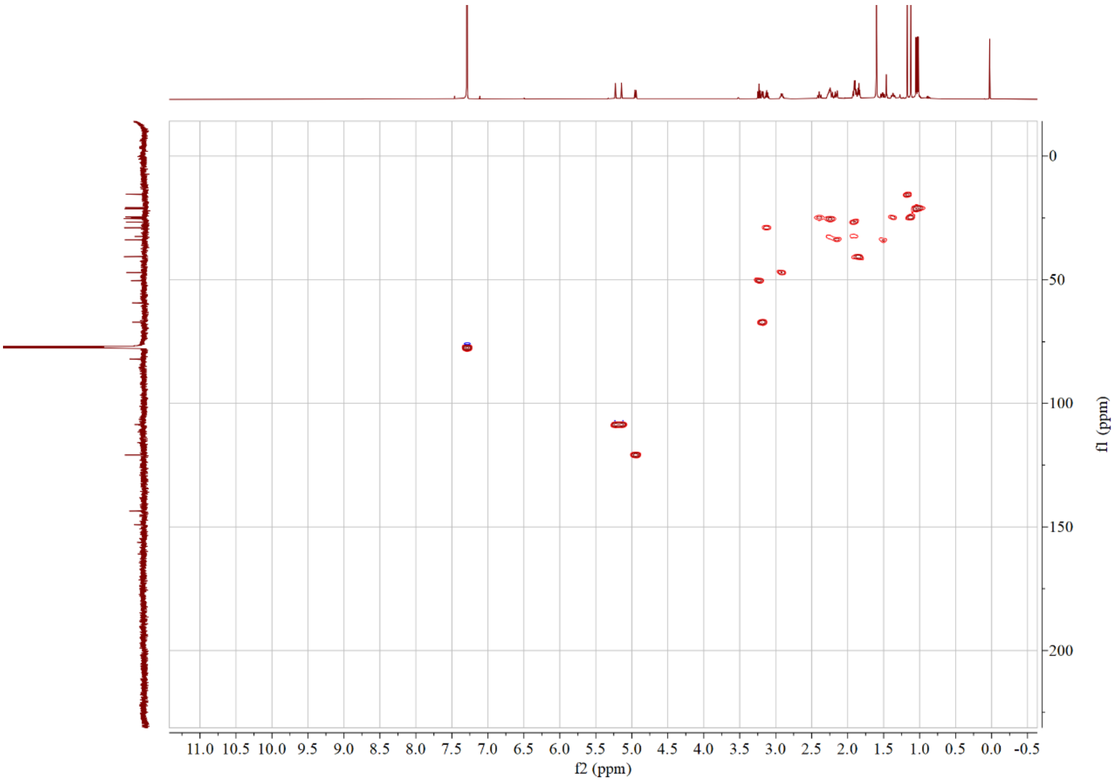

Figure S5. HSQC spectrum of **1** (600 MHz, $CDCl_3$)

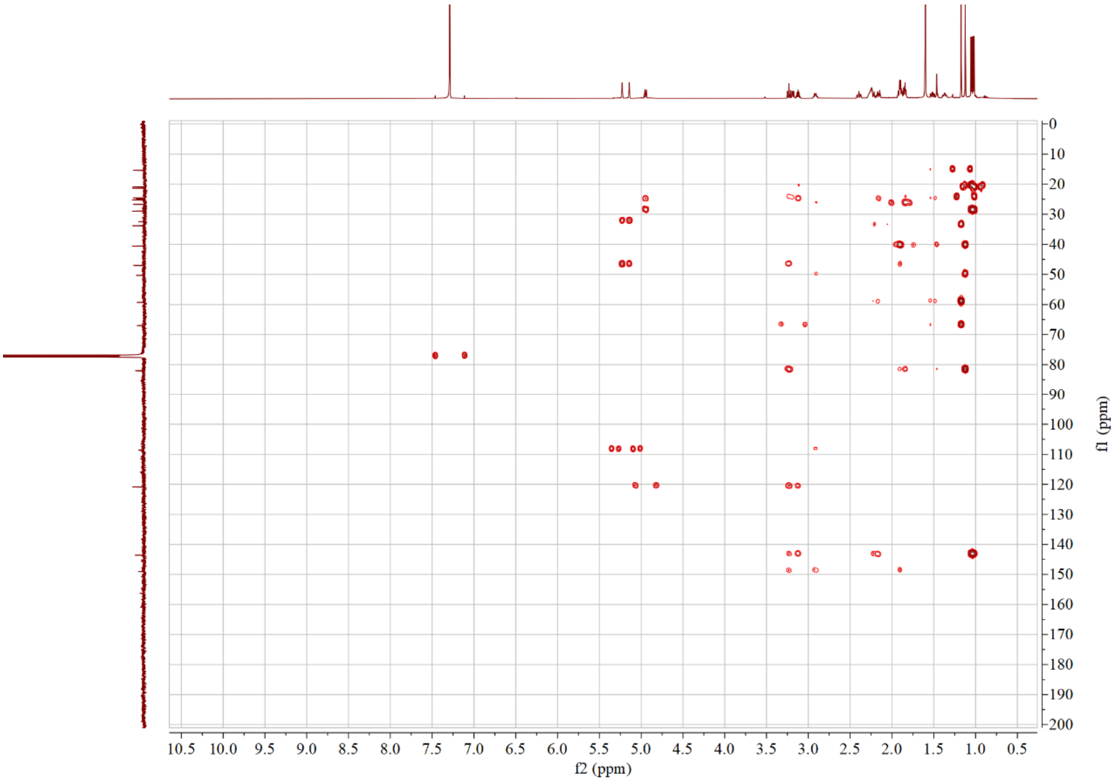

Figure S6. HMBC spectrum of **1** (600 MHz, $CDCl_3$)

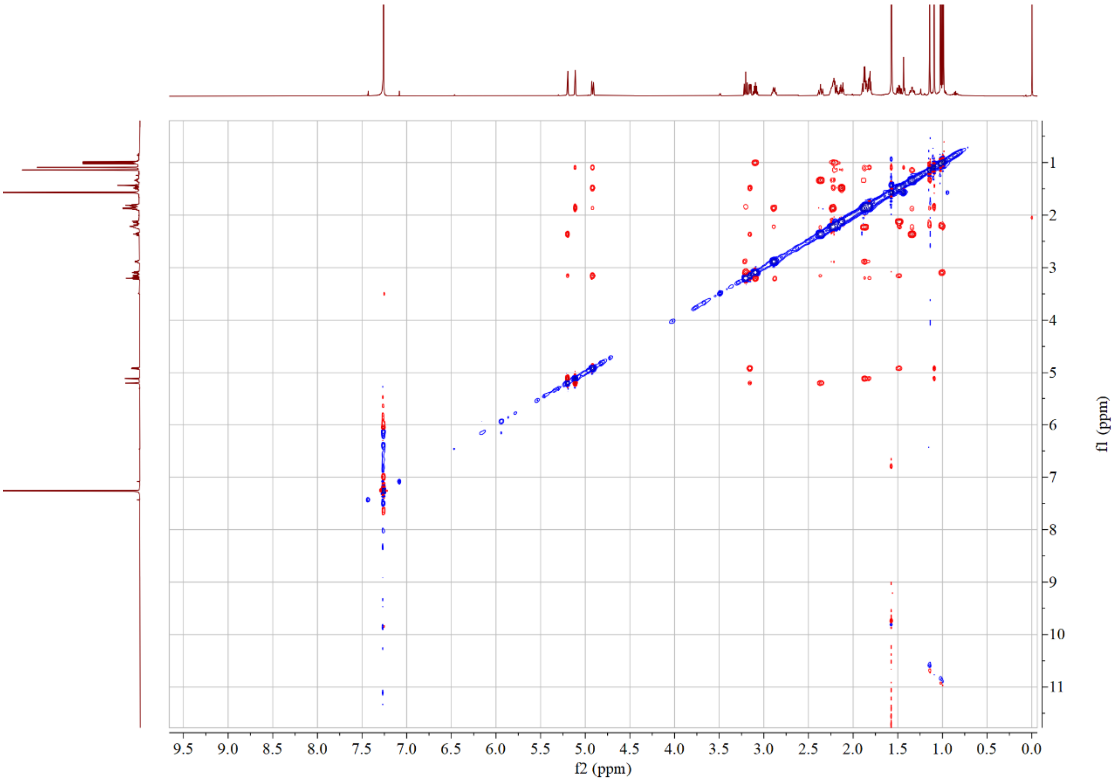

Figure S7. NOESY spectrum of **1** (600 MHz, CDCl3)

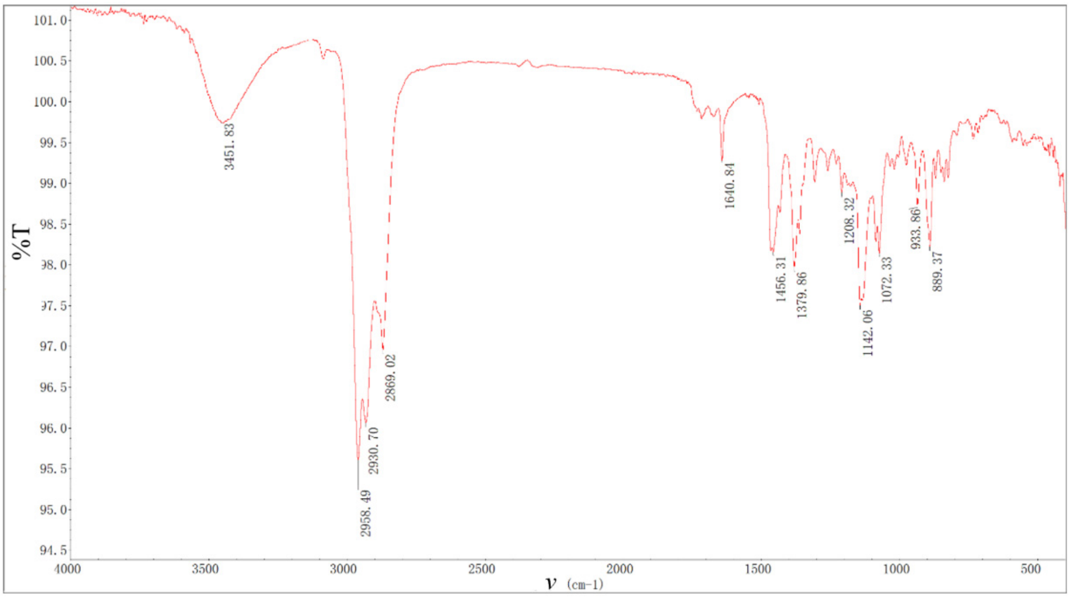

Figure S8. IR spectrum of **1**

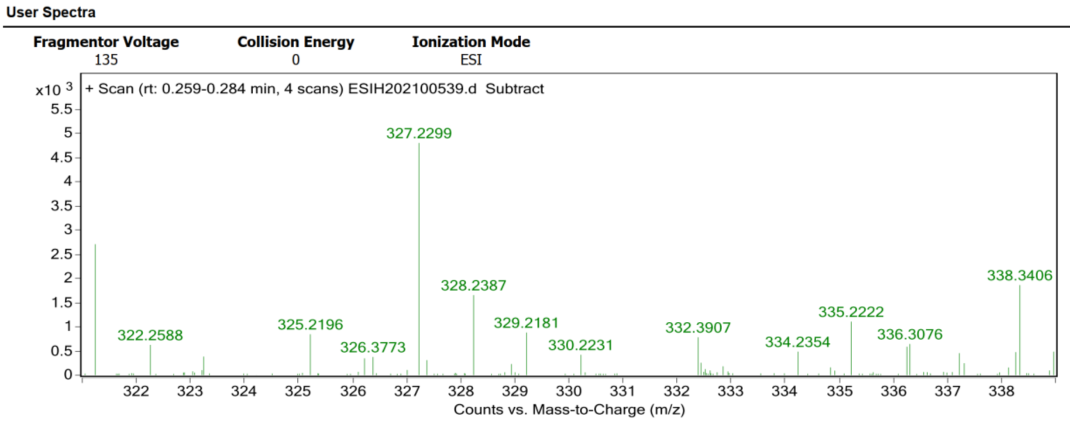

Formula Calculator Results

m/z	Calc m/z	Diff (mDa)	Diff (ppm)	Ion Formula	Ion
327.2299	327.2295	-0.49	-1.49	C20 H32 Na O2	(M+Na)+

Figure S9. HRESIMS spectrum of **1**

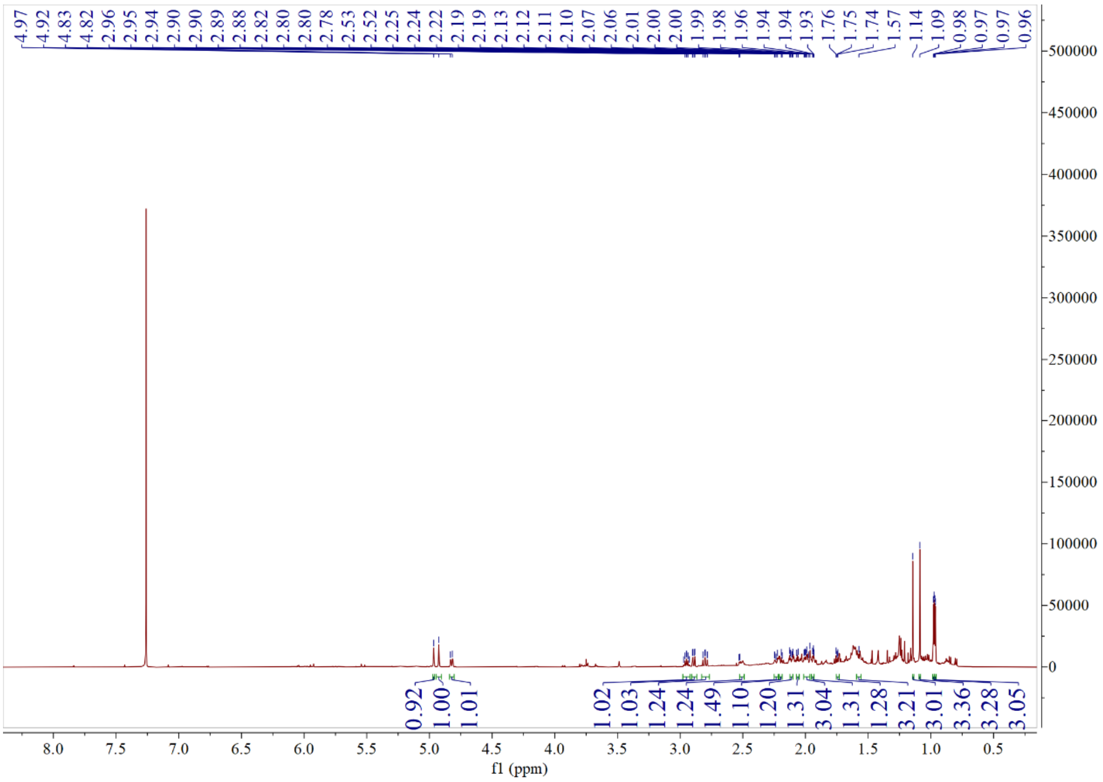

Figure S10. ^{1}H NMR spectrum of **2** (600MHz, $CDCl_3$)

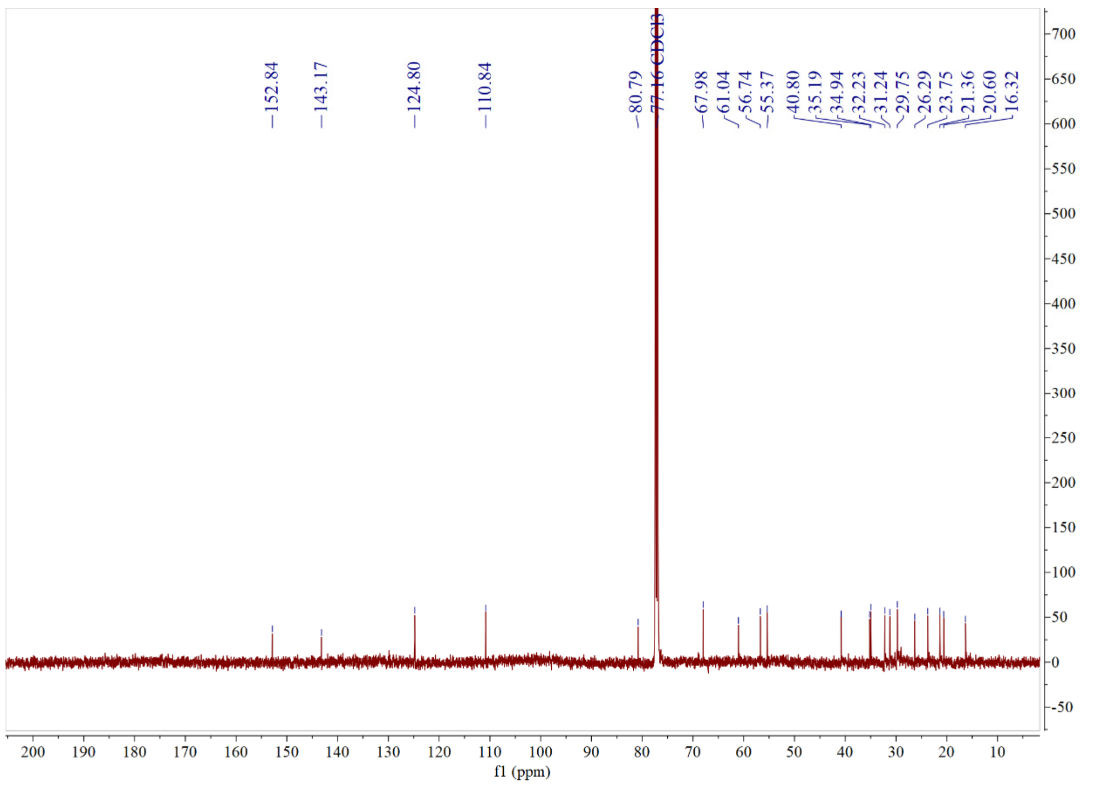

Figure S11. ^{13}C NMR spectrum of **2** (150 MHz, $CDCl_3$)

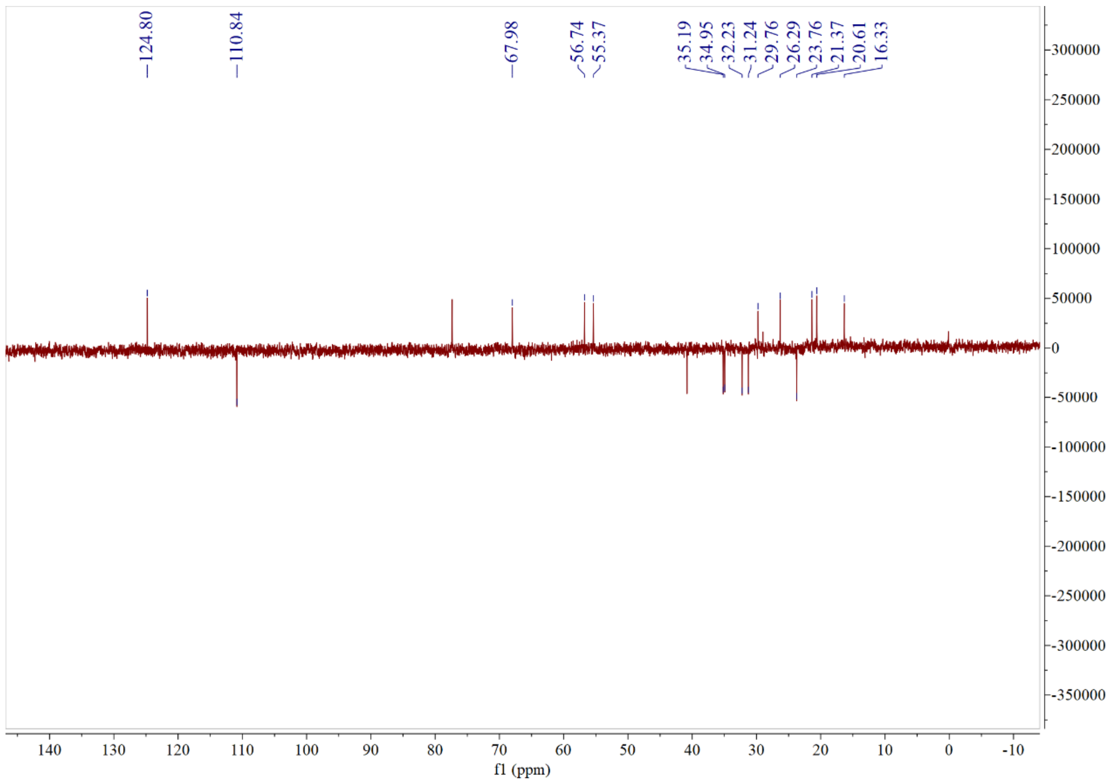

Figure S12. DEPT spectrum of **2** (150 MHz, $CDCl_3$)

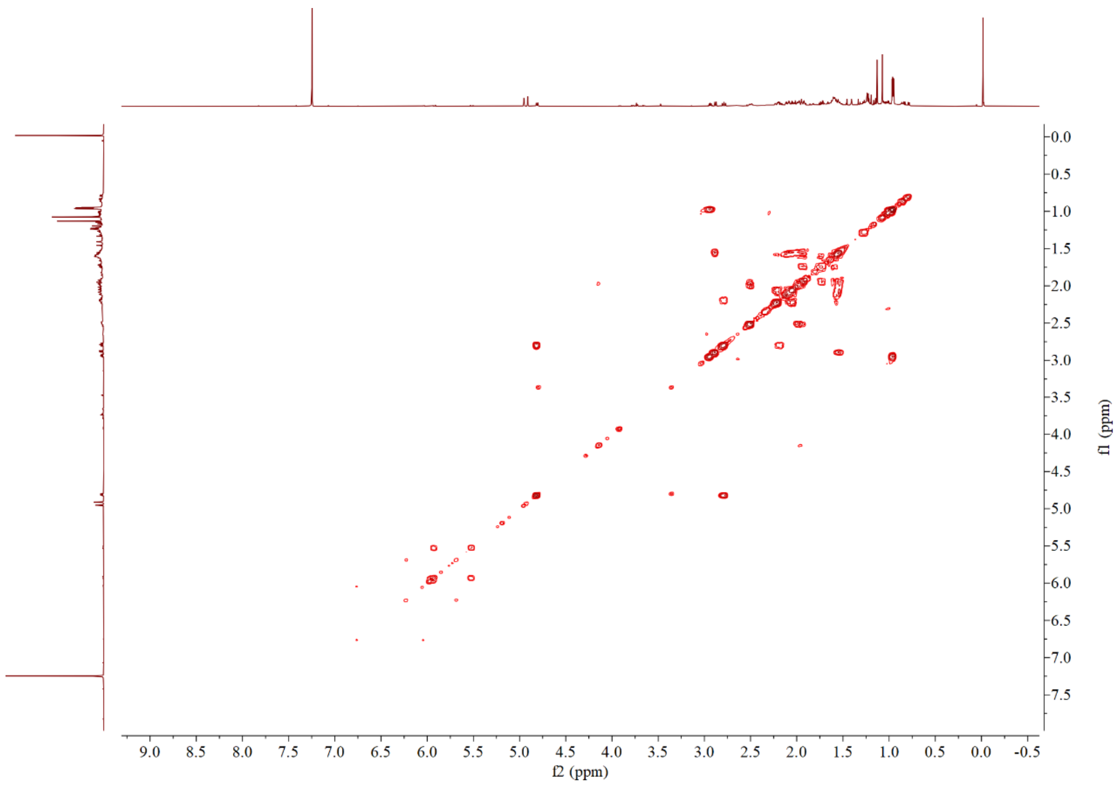

Figure S13. ^{1}H–^{1}H COSY spectrum of **2** (600 MHz, $CDCl_3$)

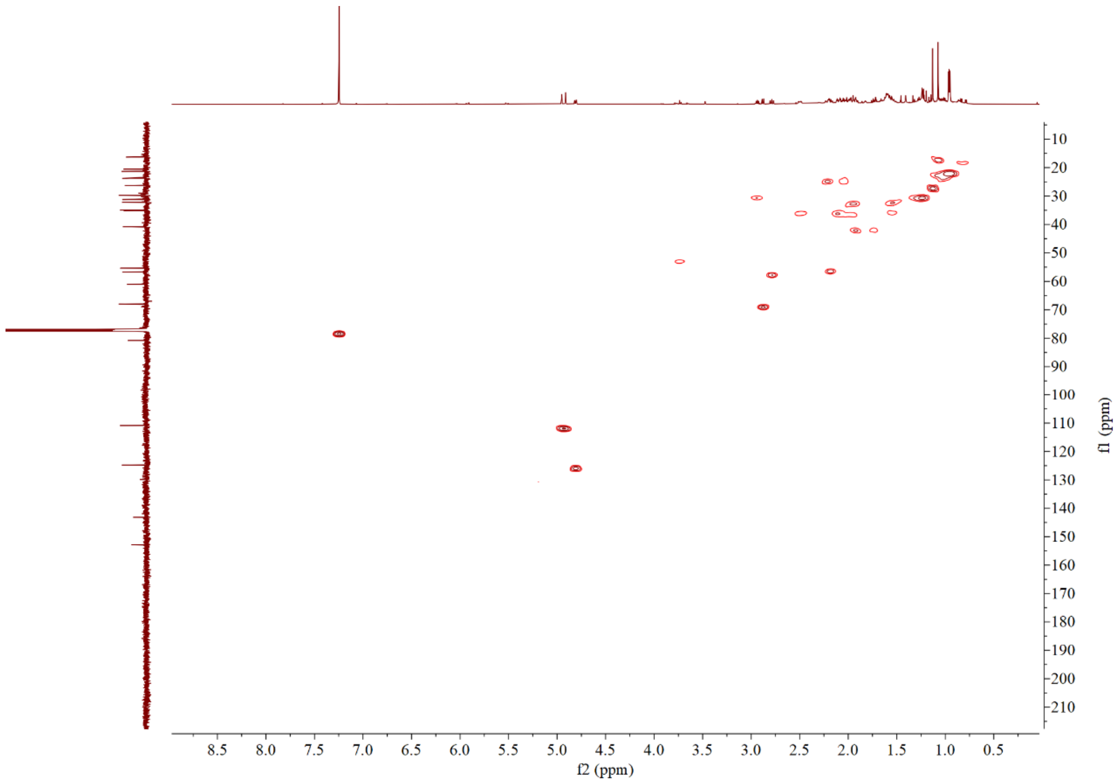

Figure S14. HSQC spectrum of **2** (600 MHz, $CDCl_3$)

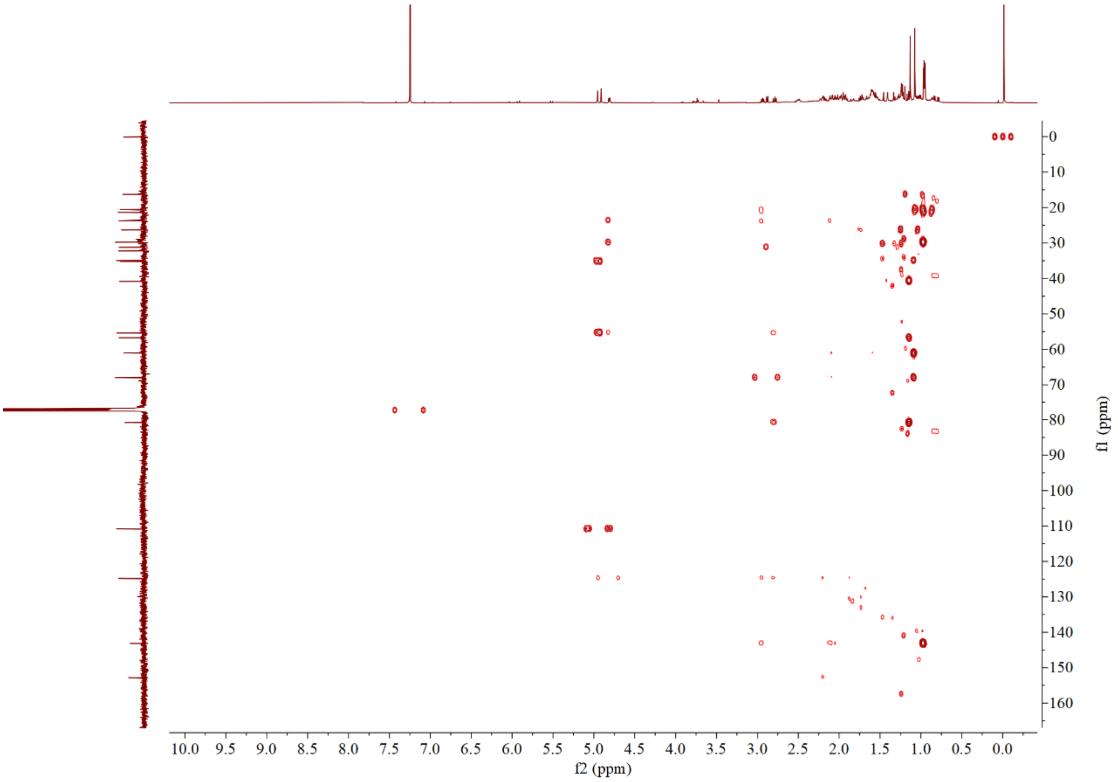

Figure S15. HMBC spectrum of **2** (600 MHz, $CDCl_3$)

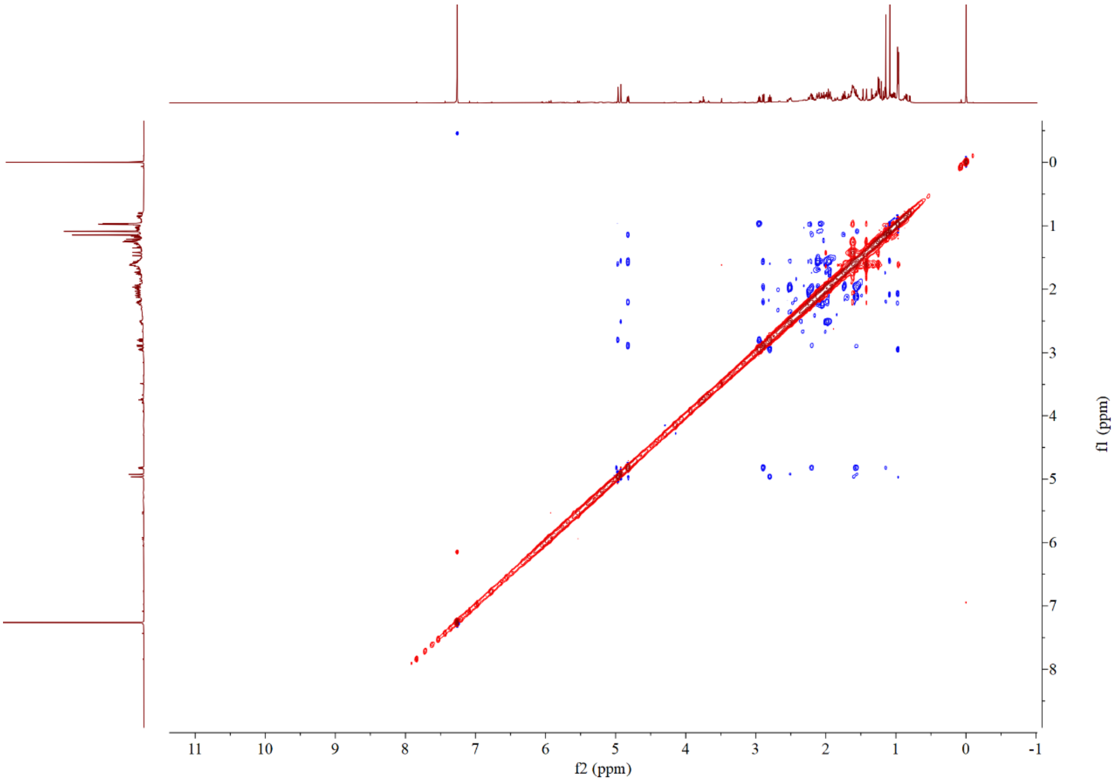

Figure S16. NOESY spectrum of **2** (600 MHz, $CDCl_3$)

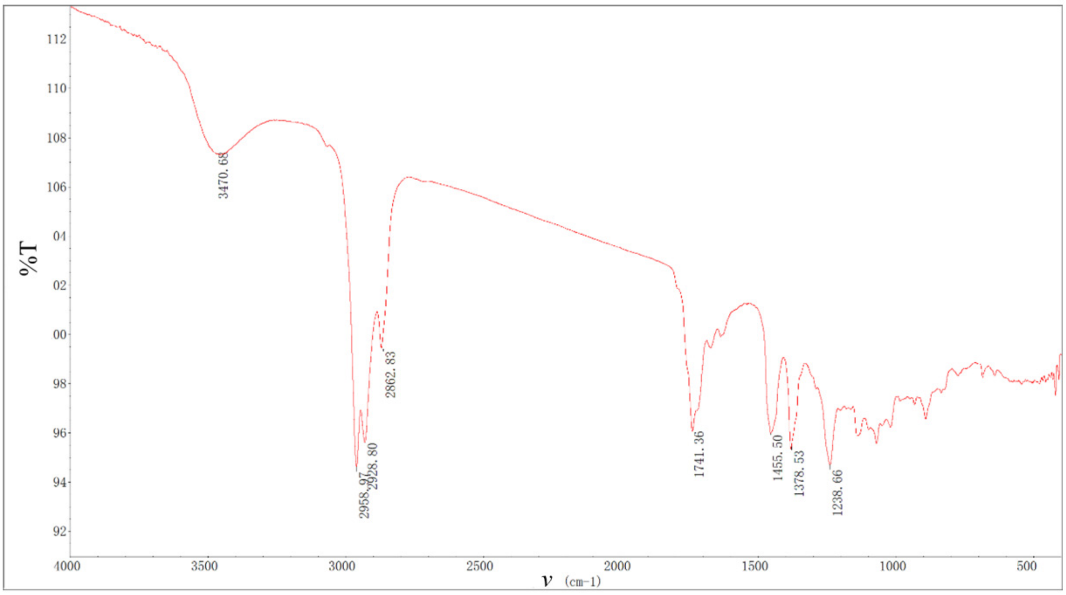

Figure S17. IR spectrum of **2**

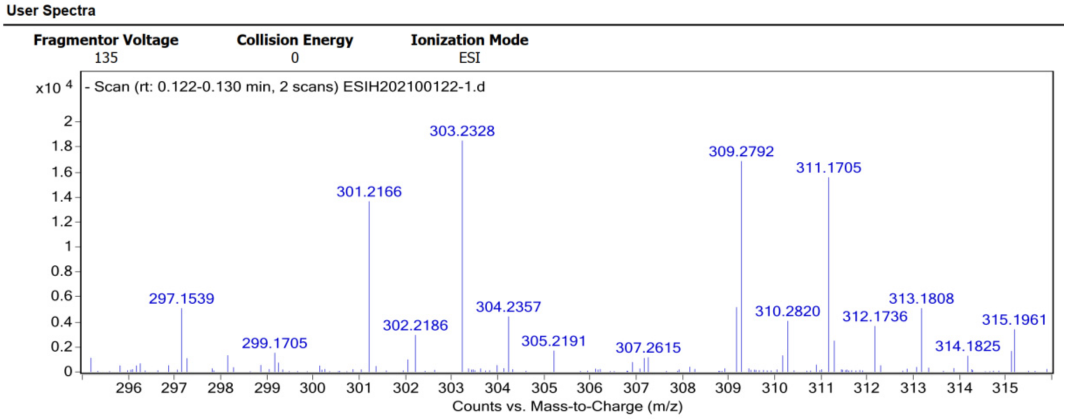

Formula Calculator Results

m/z	Calc m/z	Diff (mDa)	Diff (ppm)	Ion Formula	Ion
303.2328	303.233	0.2	0.67	C20 H31 O2	(M-H)-

Figure S18. HRESIMS spectrum of **2**

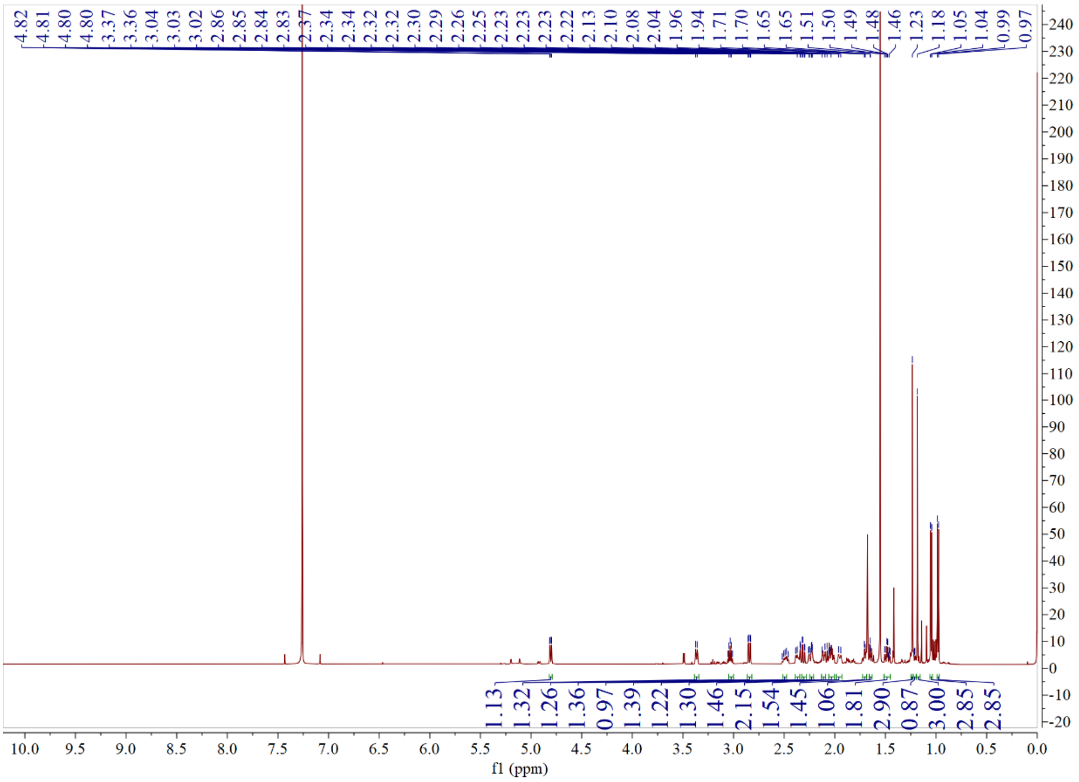

Figure S19. ^{1}H NMR spectrum of **3** (600MHz, $CDCl_3$)

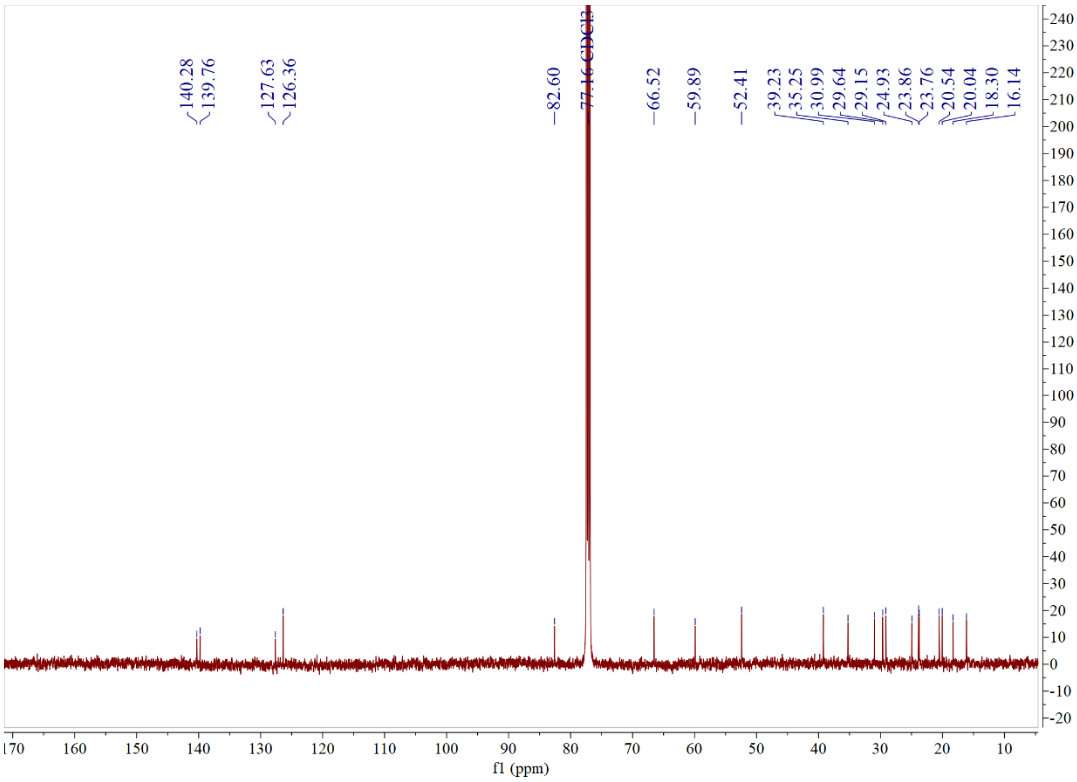

Figure S20. ^{13}C NMR spectrum of **3** (150 MHz, $CDCl_3$)

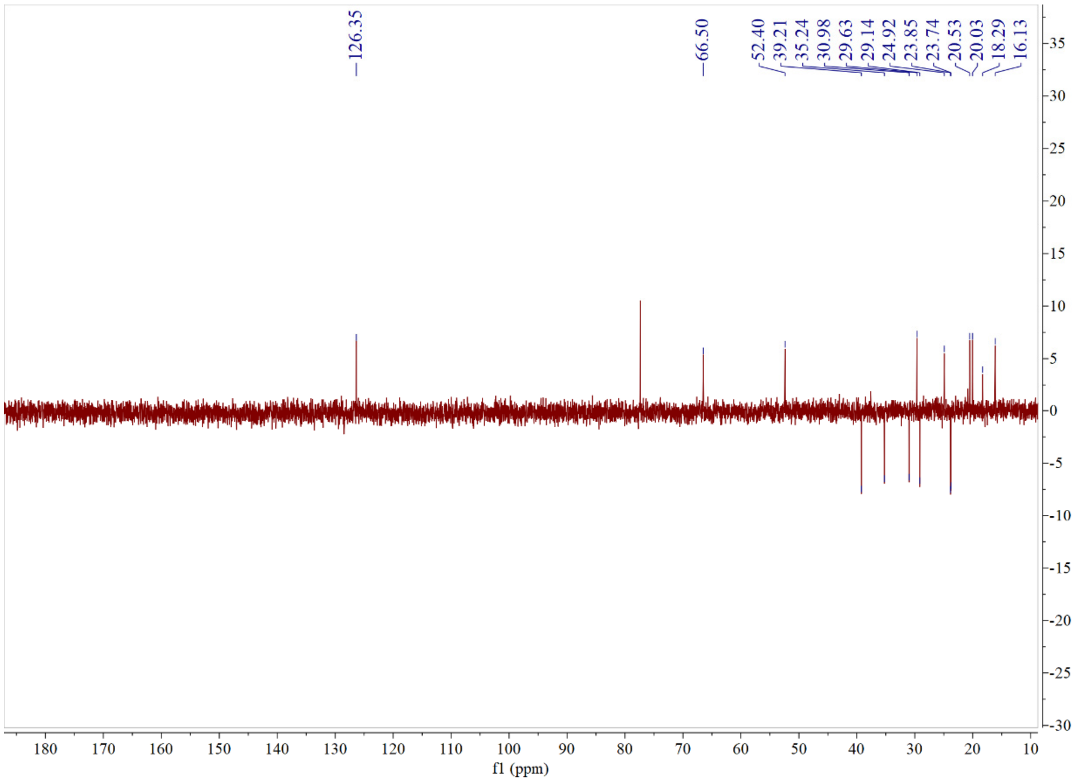

Figure S21. DEPT spectrum of **3** (150 MHz, CDCl3)

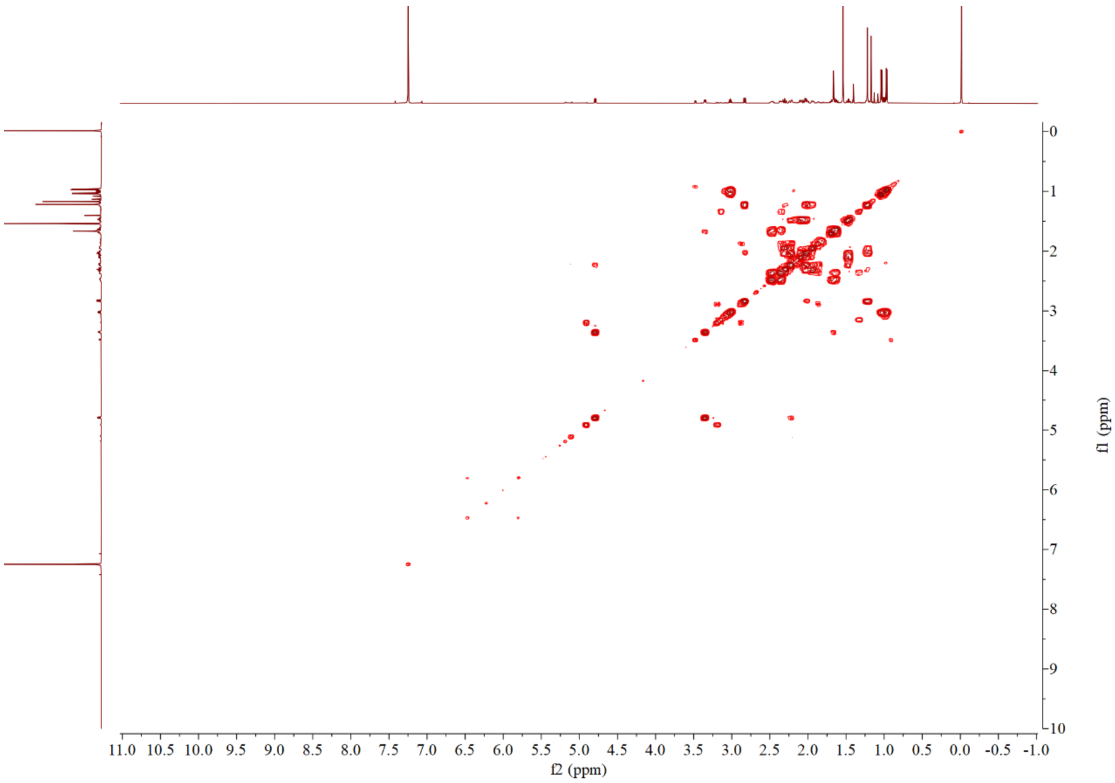

Figure S22. ¹H–¹H COSY spectrum of **3** (600 MHz, CDCl3)

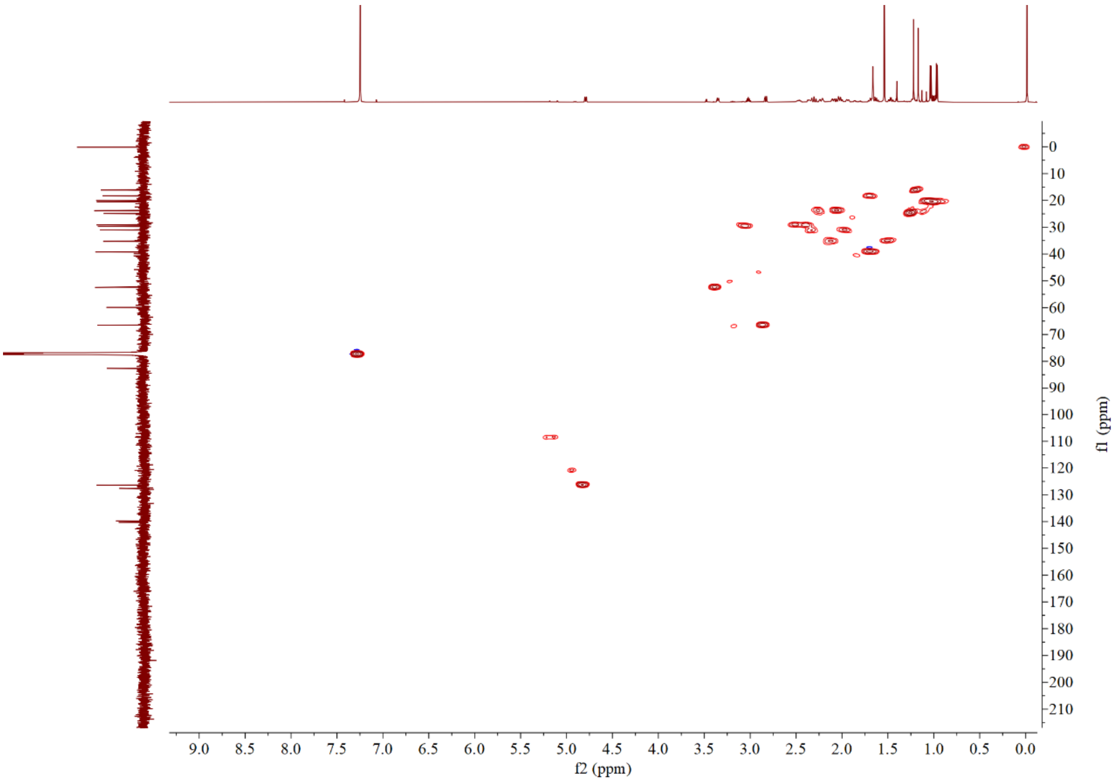

Figure S23. HSQC spectrum of **3** (600MHz, $CDCl_3$)

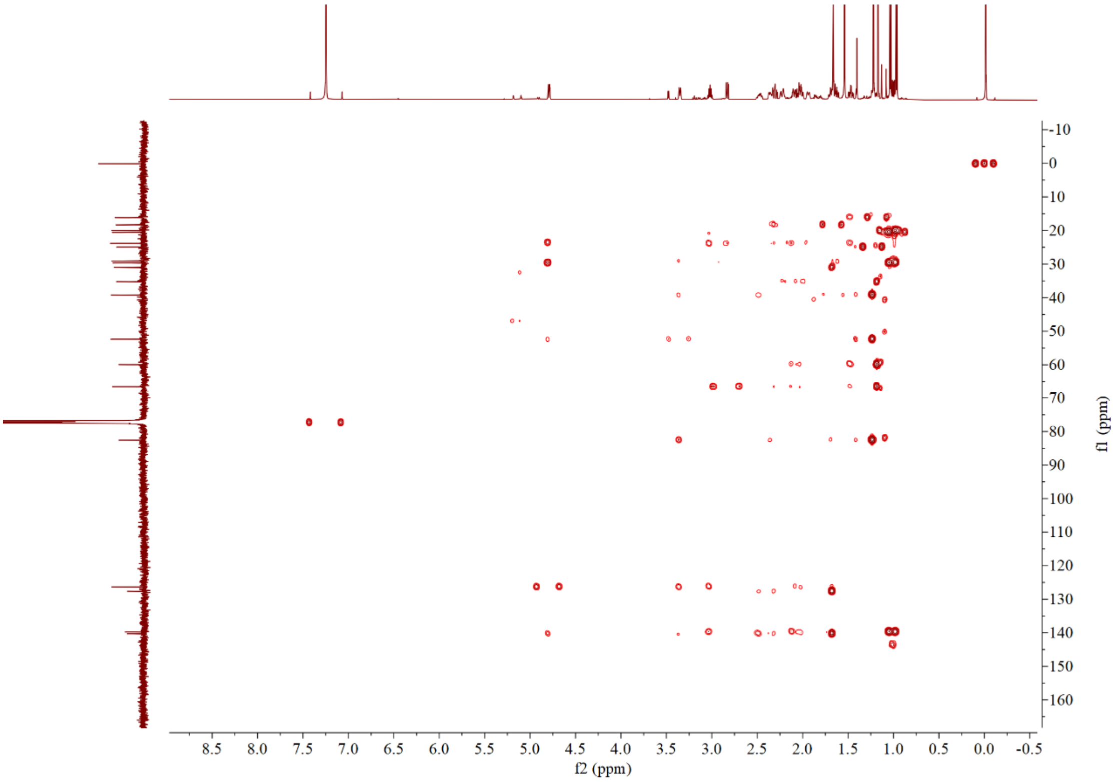

Figure S24. HMBC spectrum of **3** (600 MHz, $CDCl_3$)

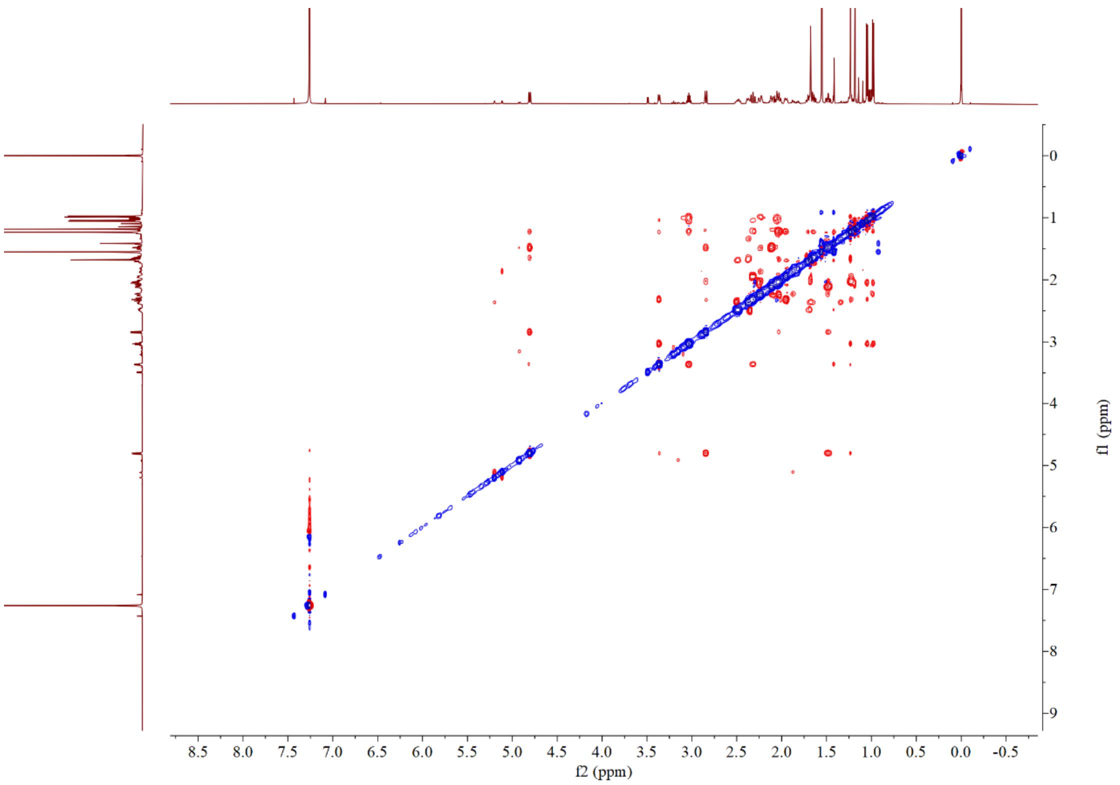

Figure S25. NOEY spectrum of **3** (600MHz, $CDCl_3$)

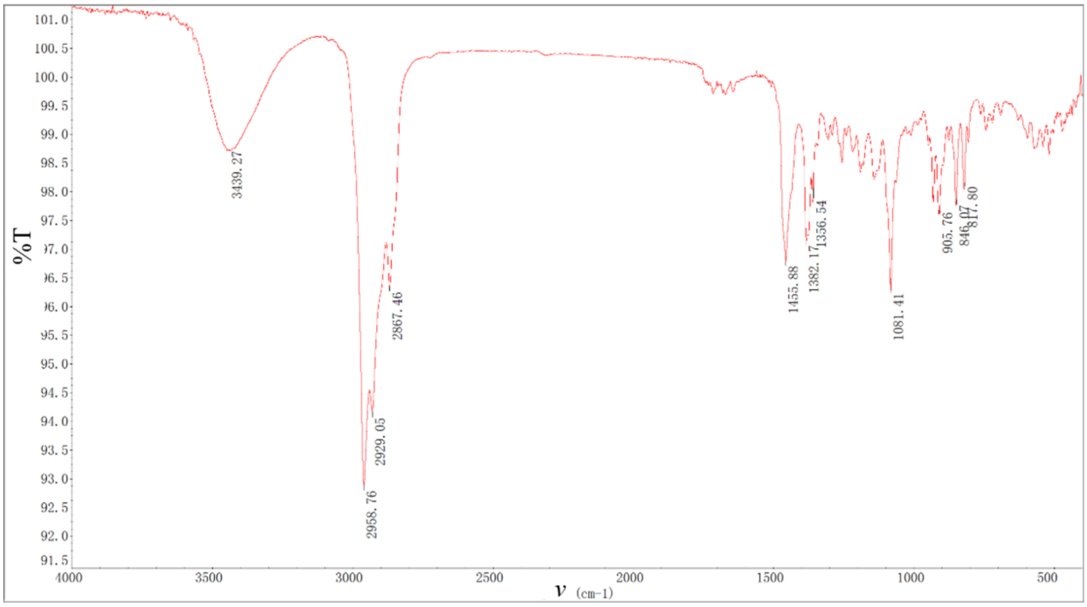

Figure S26. IR spectrum of **3**

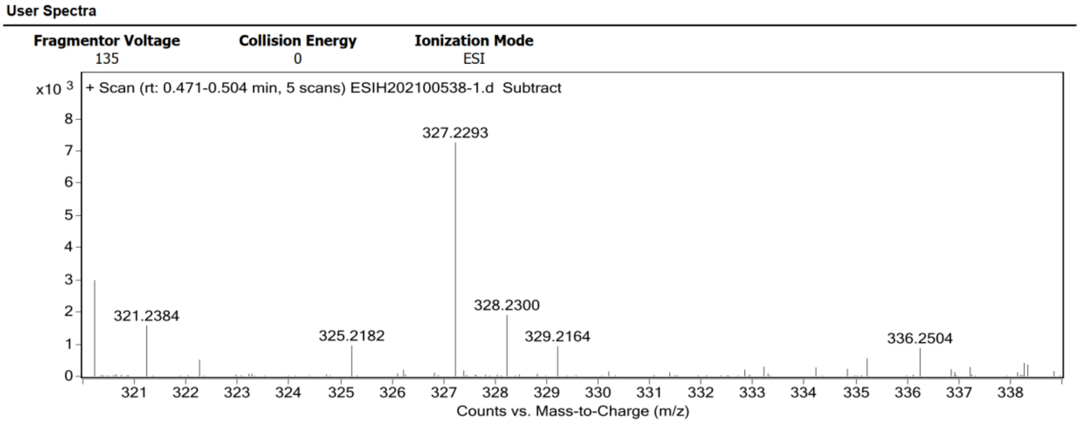

Formula Calculator Results

m/z	Calc m/z	Diff (mDa)	Diff (ppm)	Ion Formula	Ion
327.2293	327.2295	0.15	0.45	C20 H32 Na O2	(M+Na)+

Figure S27. HRESIMR spectrum of **3**

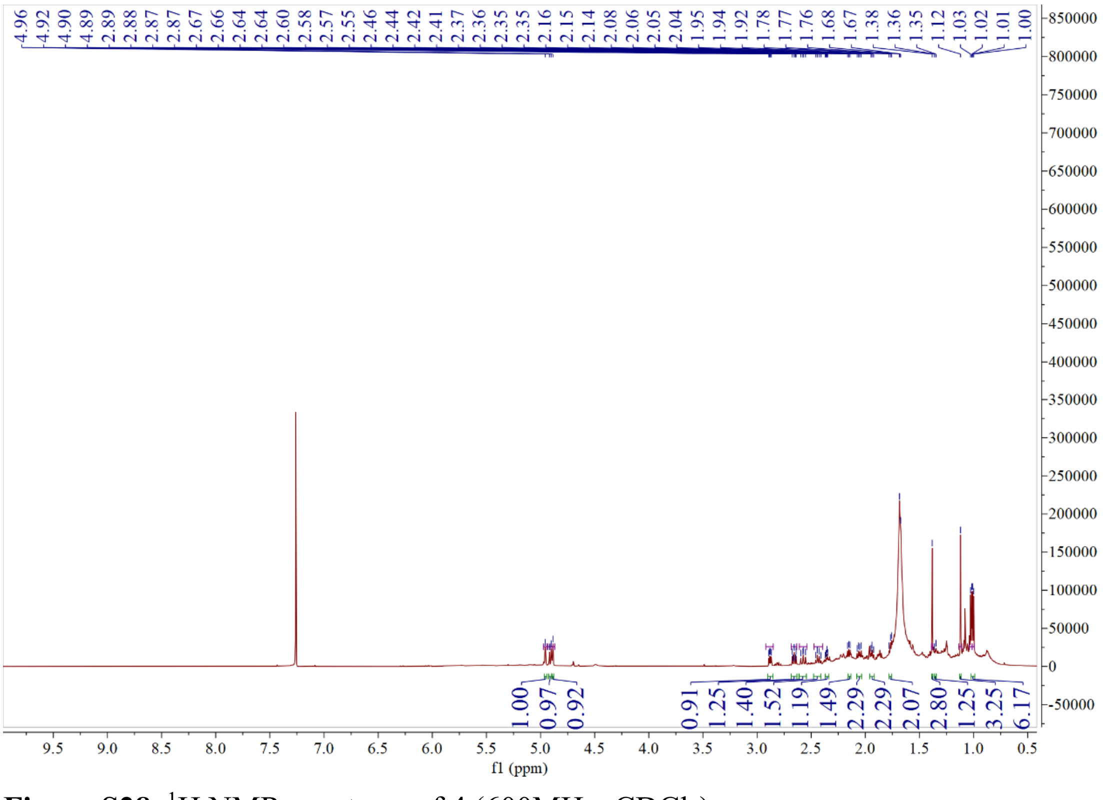

Figure S28. ^{1}H NMR spectrum of **4** (600MHz, $CDCl_3$)

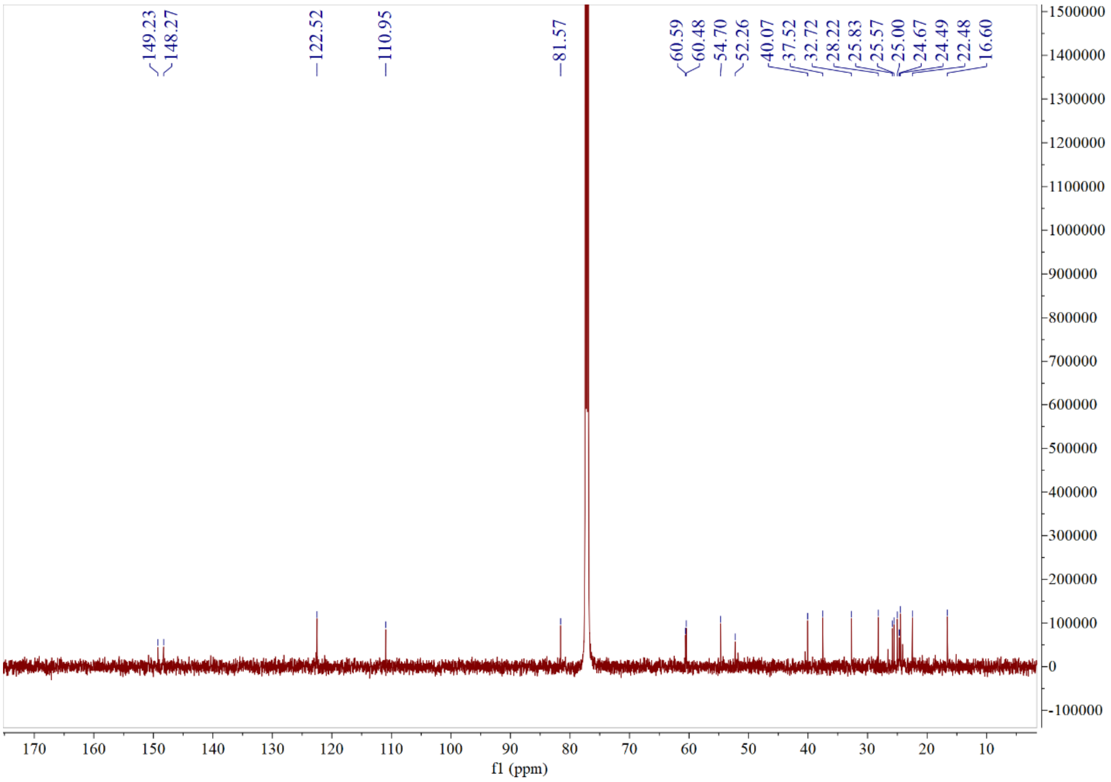

Figure S29. ^{13}C NMR spectrum of **4** (150 MHz, $CDCl_3$)

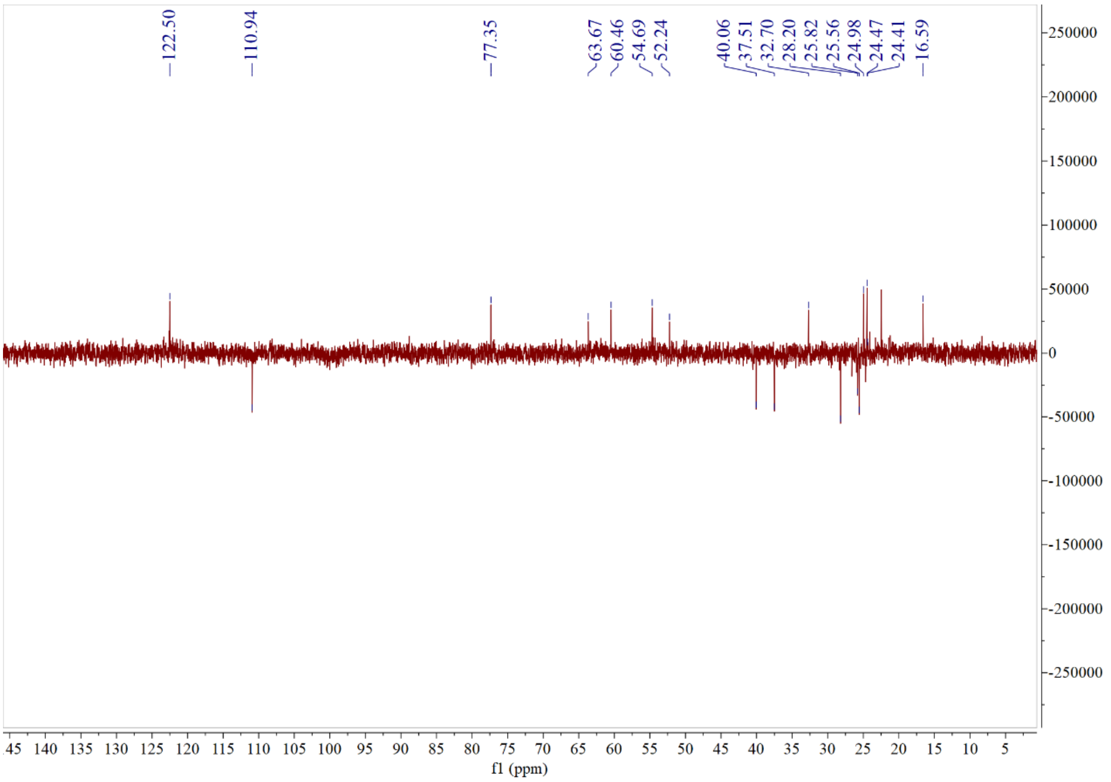

Figure S30. DEPT spectrum of **4** (150 MHz, $CDCl_3$)

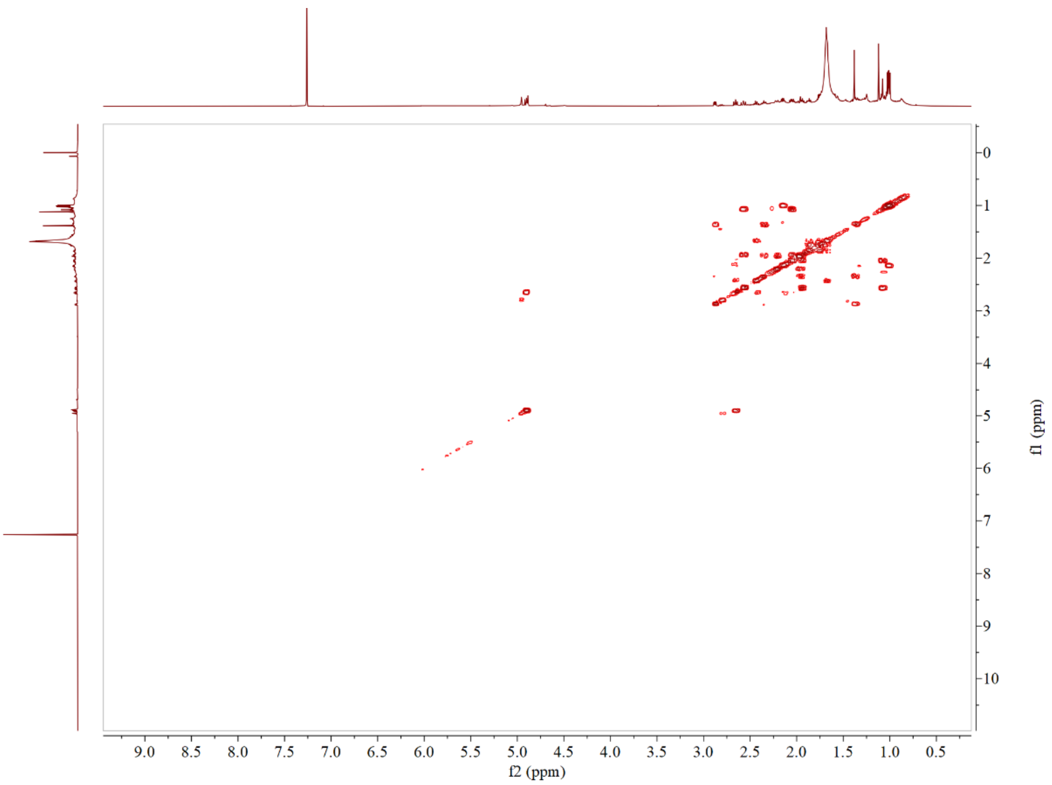

Figure S31. ^{1}H–^{1}H COSY spectrum of **4** (600 MHz, $CDCl_3$)

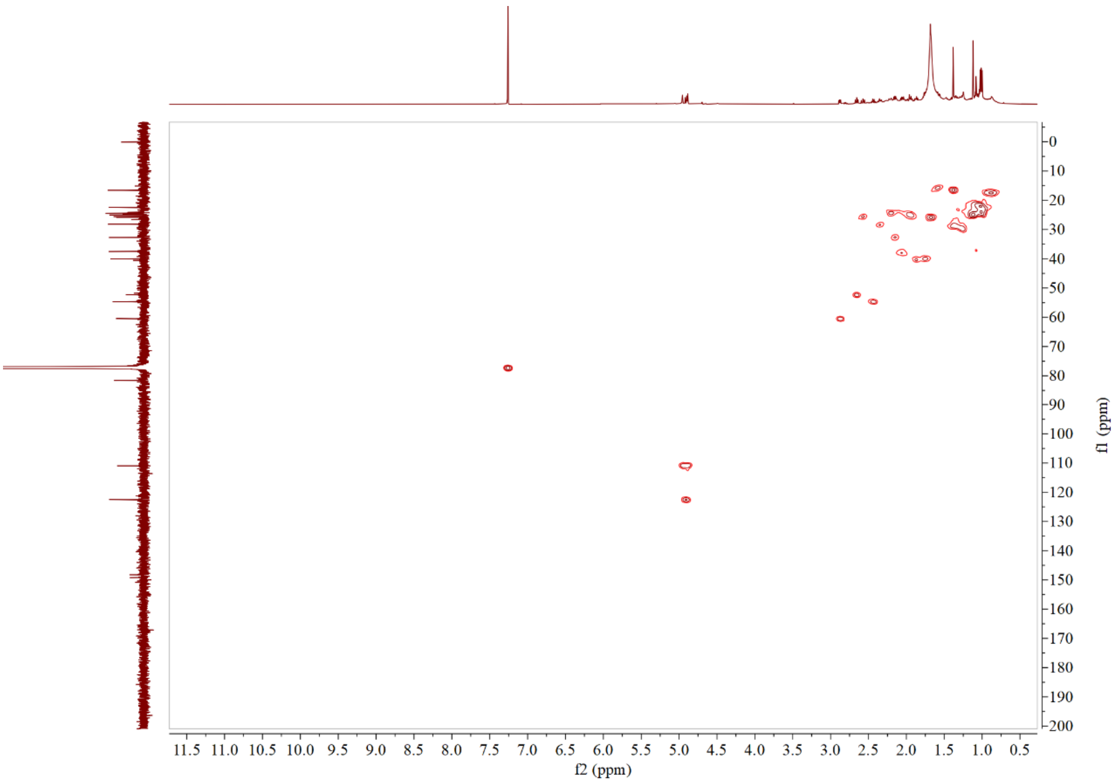

Figure S32. HSQC spectrum of **4** (600 MHz, $CDCl_3$)

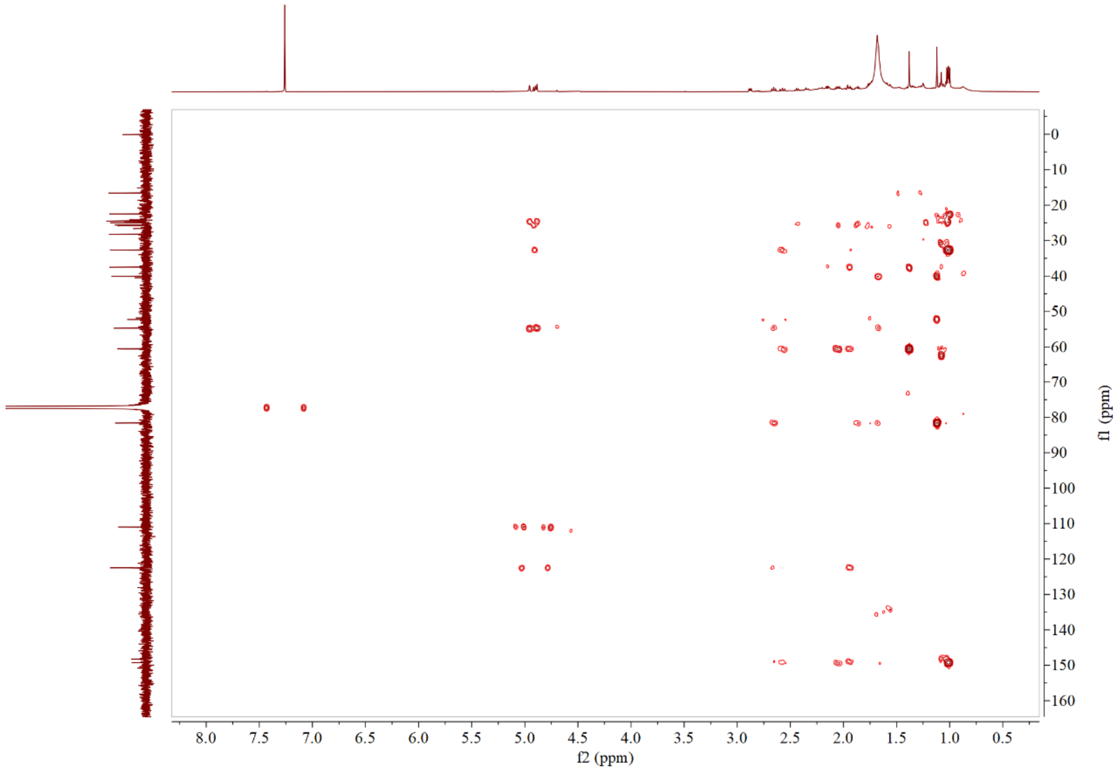

Figure S33. HMBC spectrum of **4** (600 MHz, $CDCl_3$)

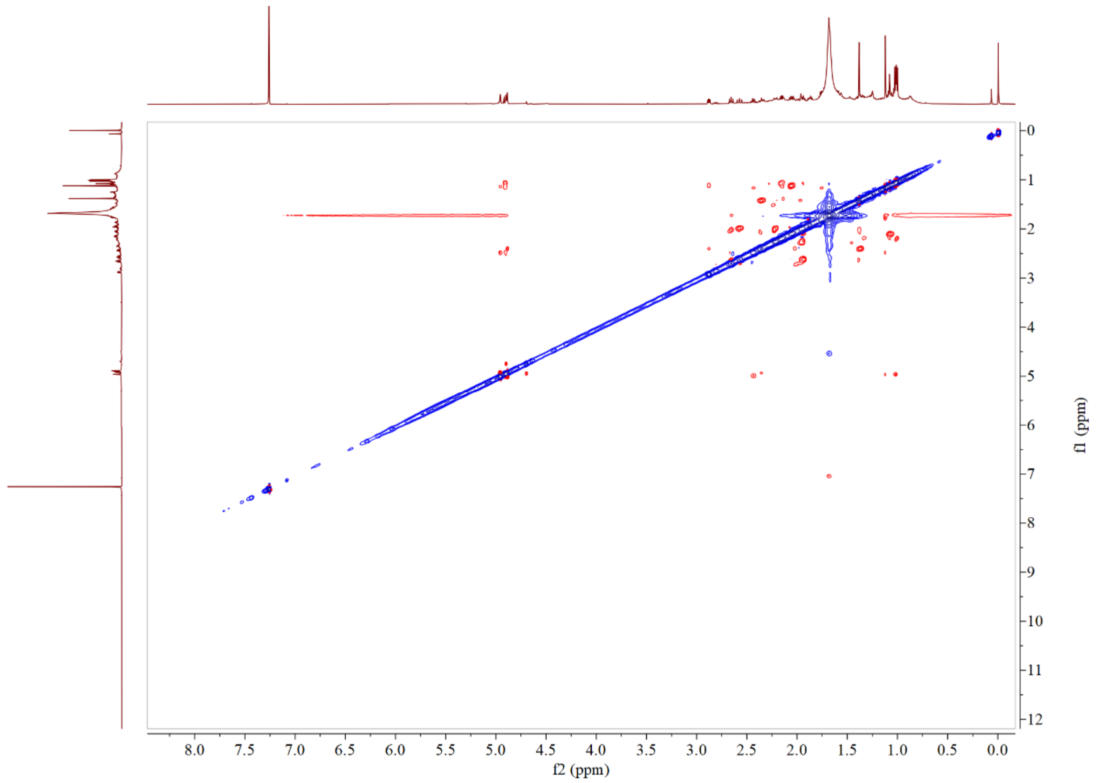

Figure S34. NOESY spectrum of **4** (600 MHz, $CDCl_3$)

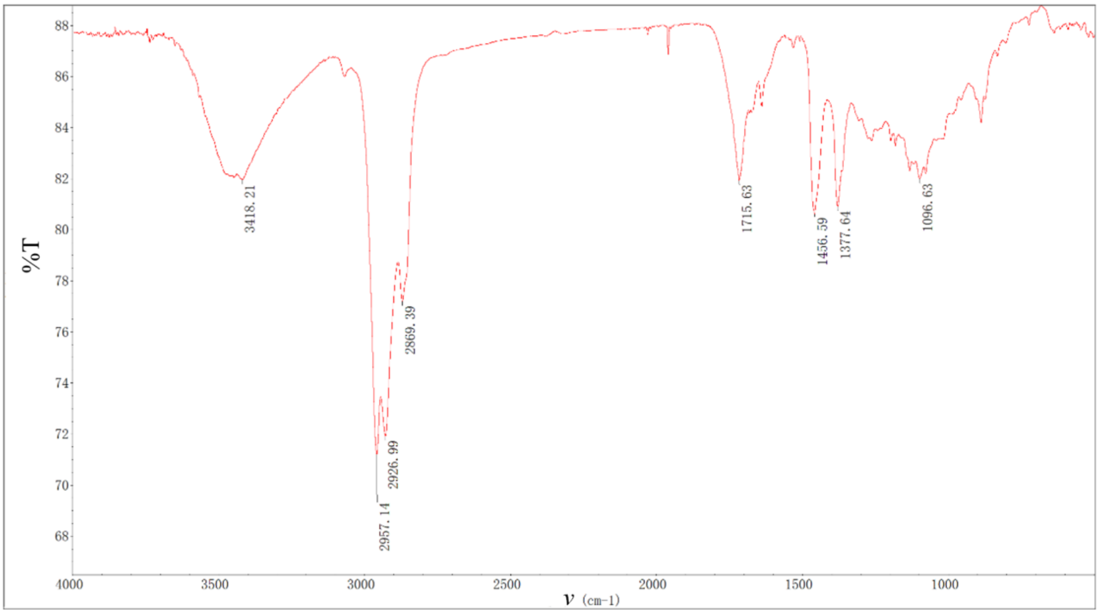

Figure S35. IR spectrum of **4**

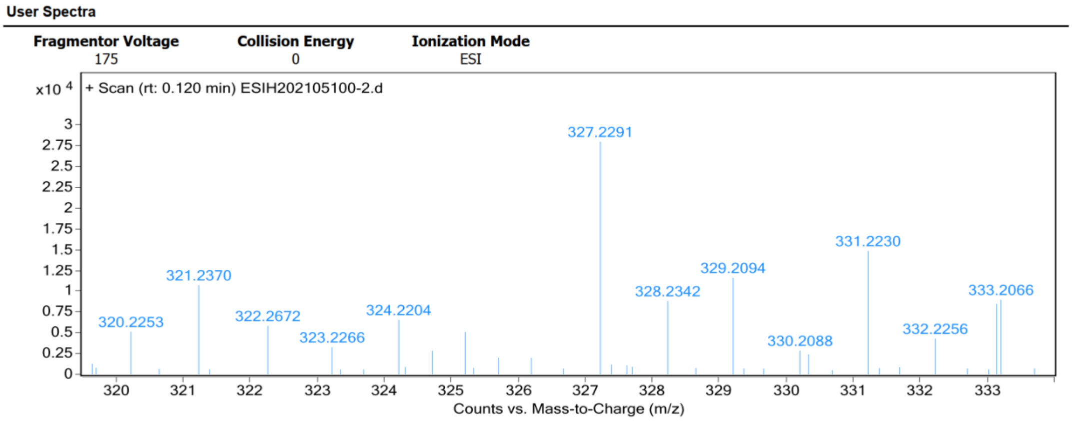

Formula Calculator Results

m/z	Calc m/z	Diff (mDa)	Diff (ppm)	Ion Formula	Ion
327.2291	327.2295	0.3	0.93	C20 H32 Na O2	(M+Na)+

Figure S36. HRESIMR spectrum of **4**

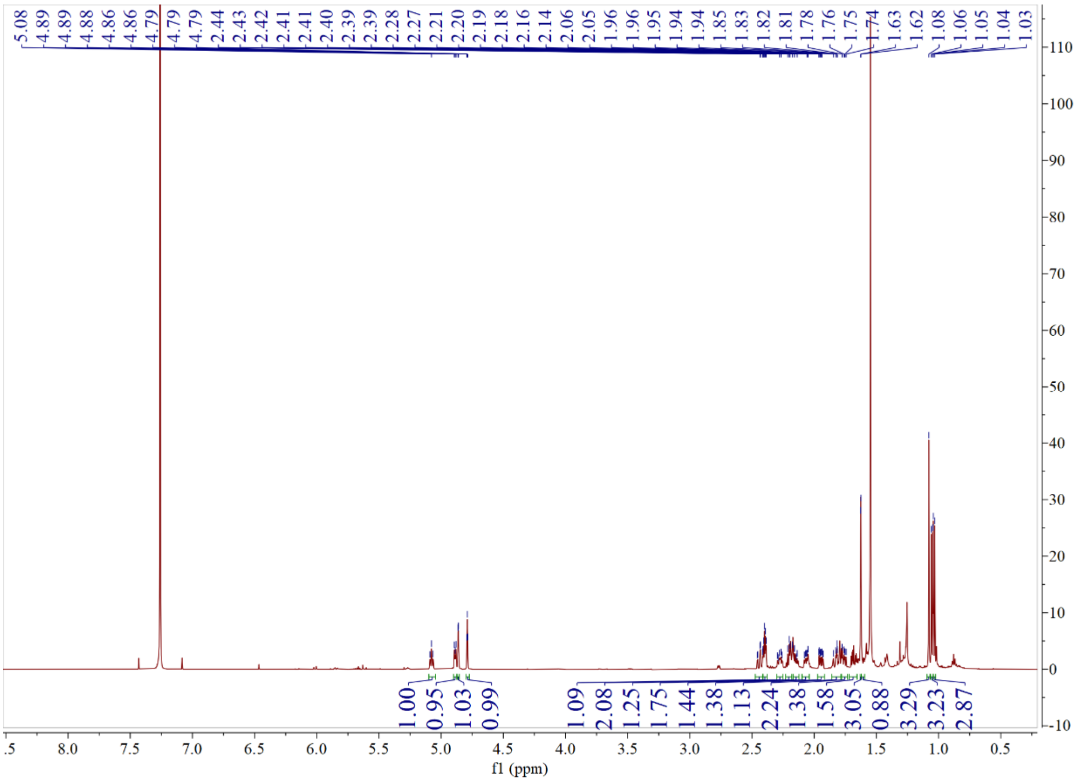

Figure S37. ^{1}H NMR spectrum of **5** (600MHz, $CDCl_3$)

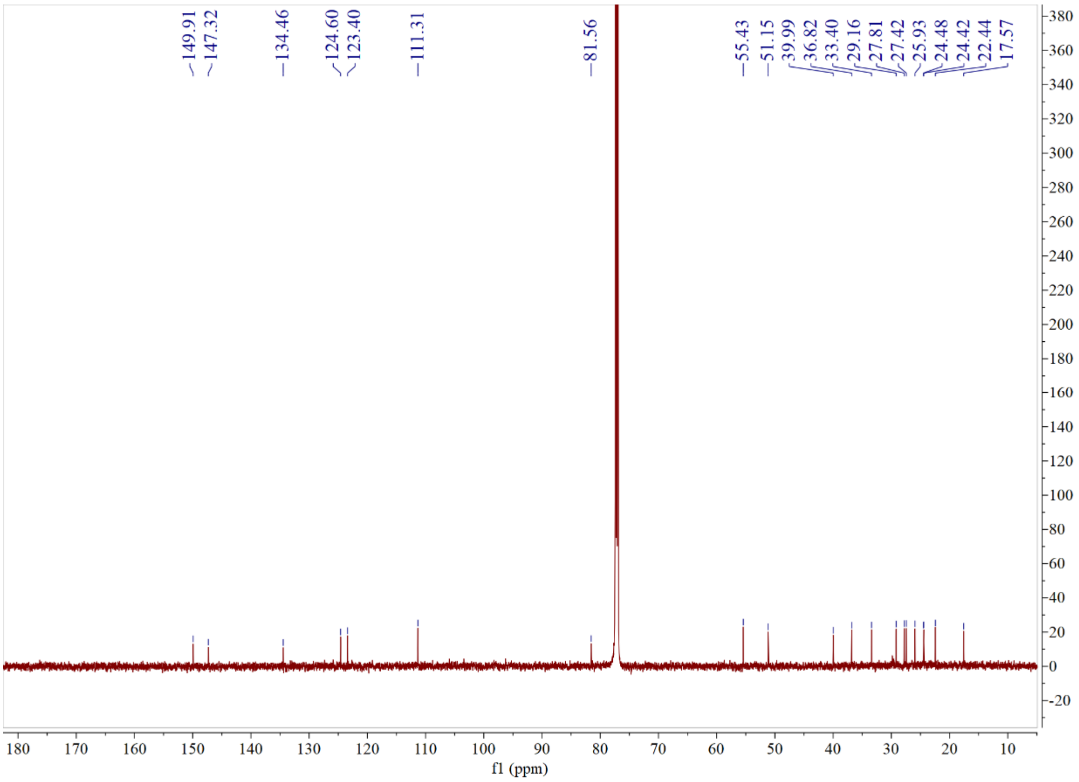

Figure S38. ^{13}C NMR spectrum of **5** (150 MHz, $CDCl_3$)

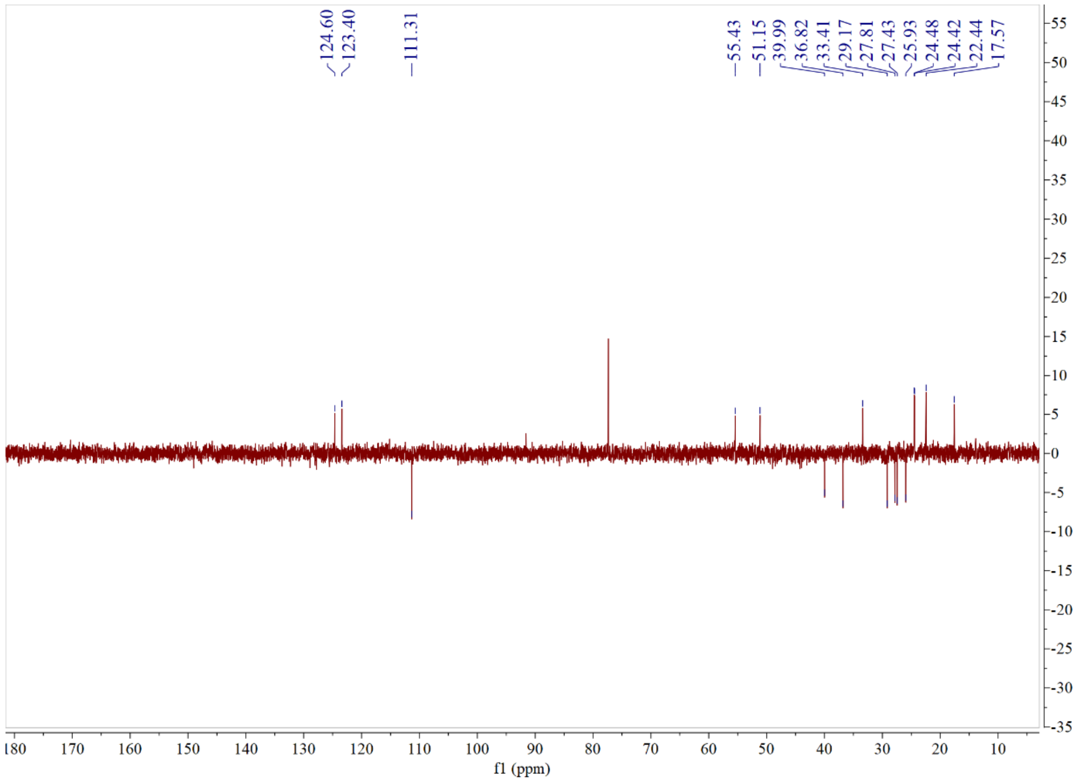

Figure S39. DEPT spectrum of **5** (150 MHz, $CDCl_3$)

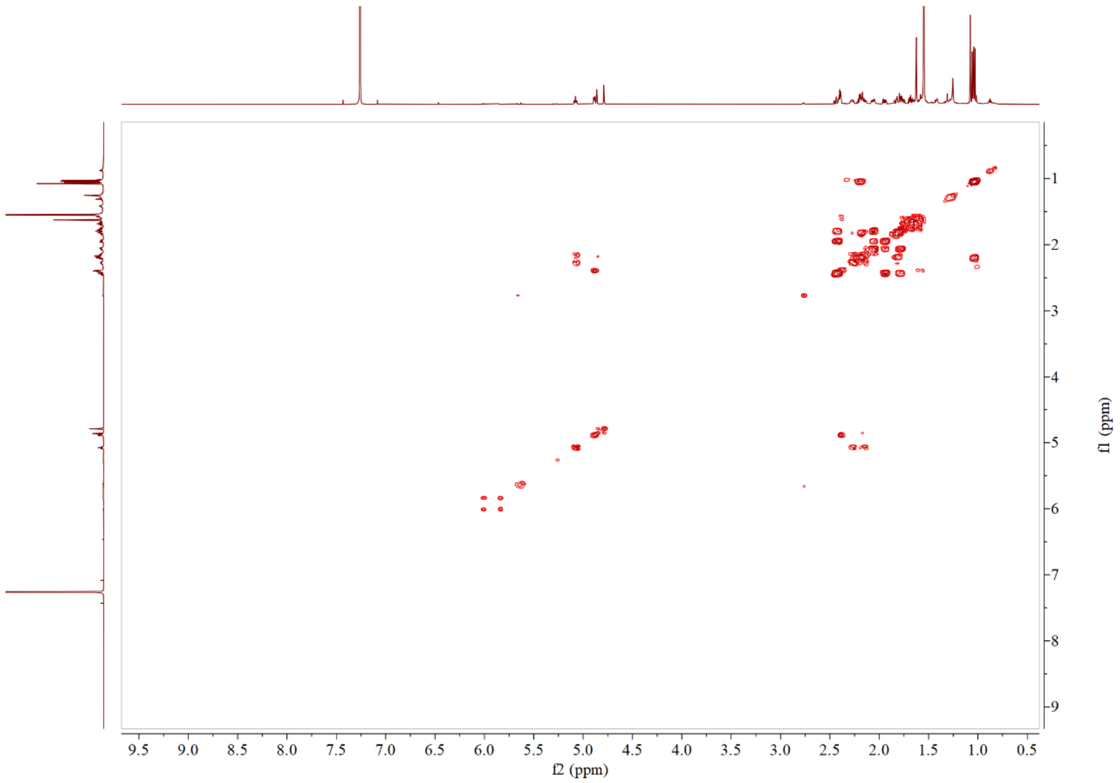

Figure S40. ^1H–^1H COSY spectrum of **5** (600 MHz, $CDCl_3$)

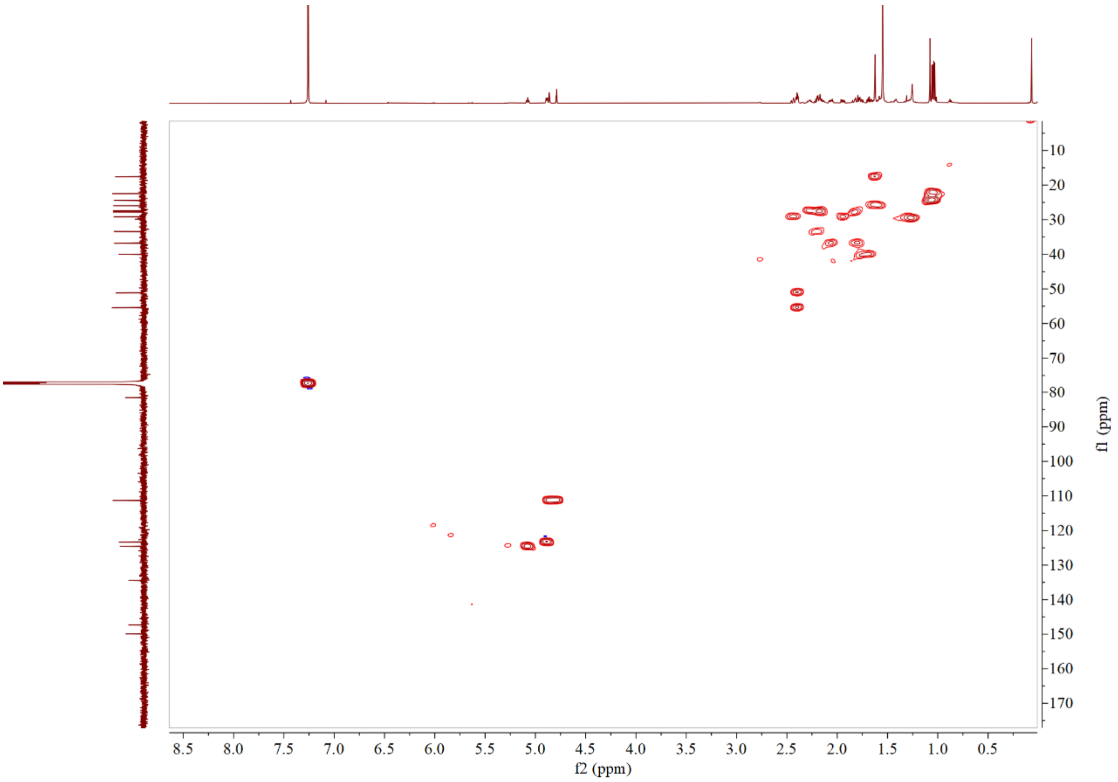

Figure S41. HSQC spectrum of **5** (600 MHz, CDCl3)

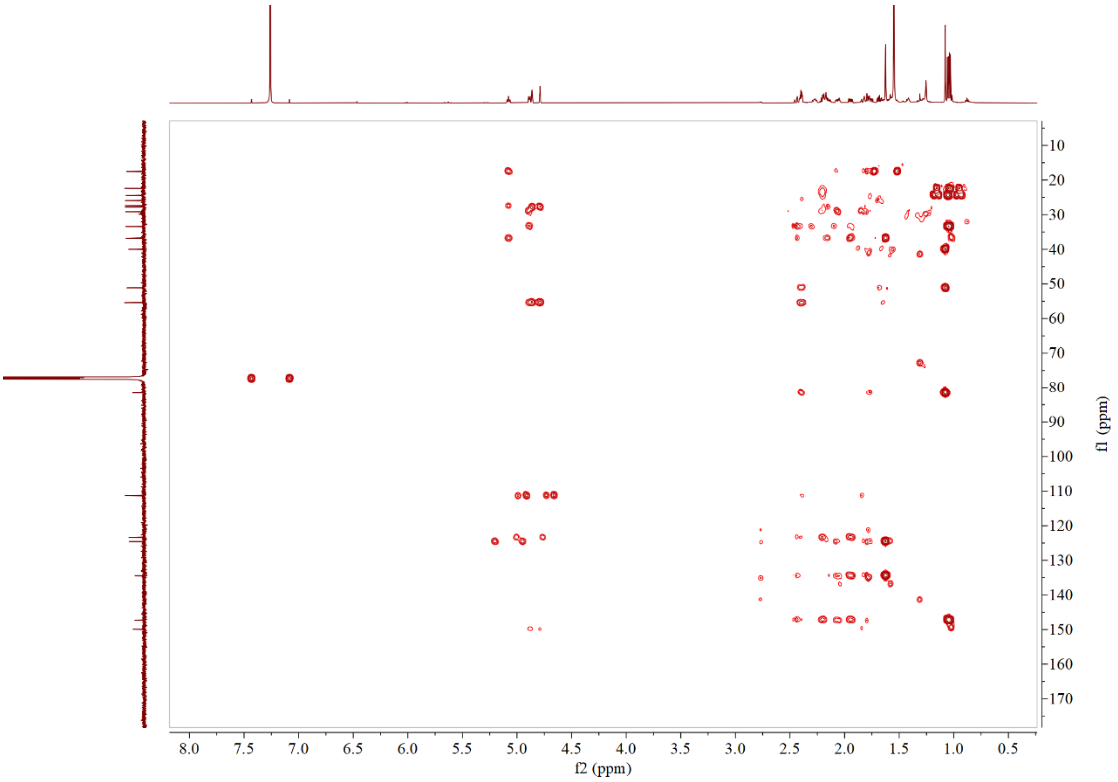

Figure S42. HMBC spectrum of **5** (600 MHz, CDCl3)

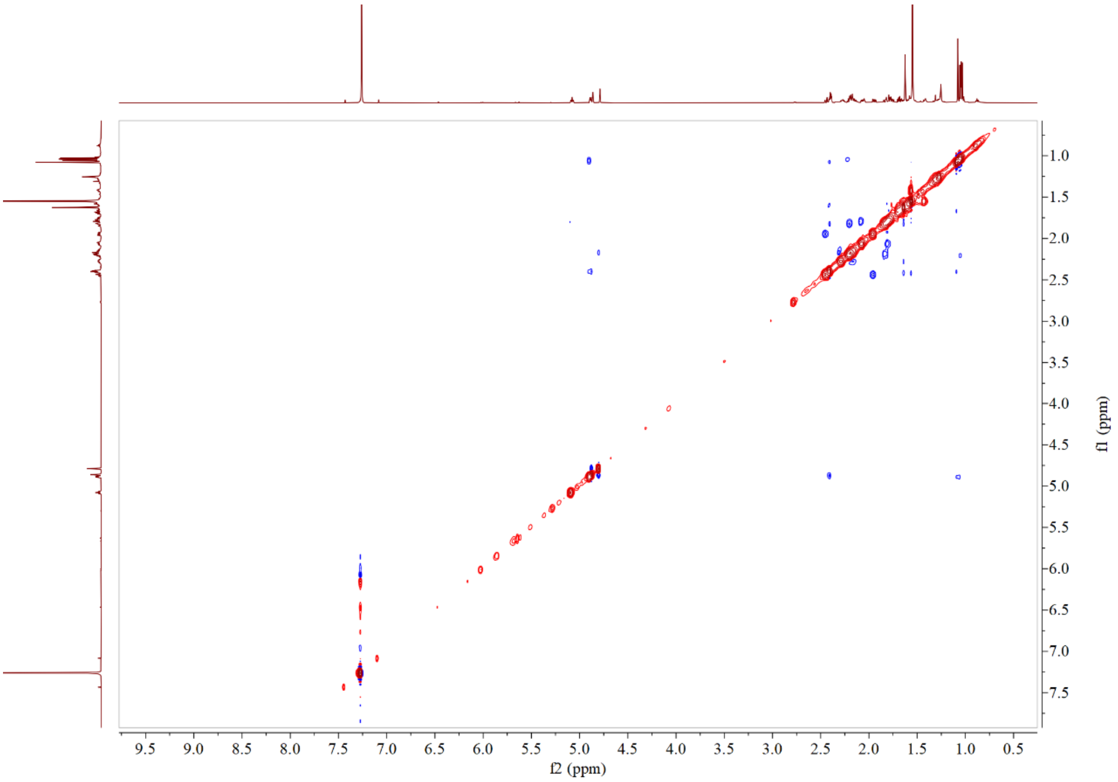

Figure S43. NOESY spectrum of **5** (600 MHz, $CDCl_3$)

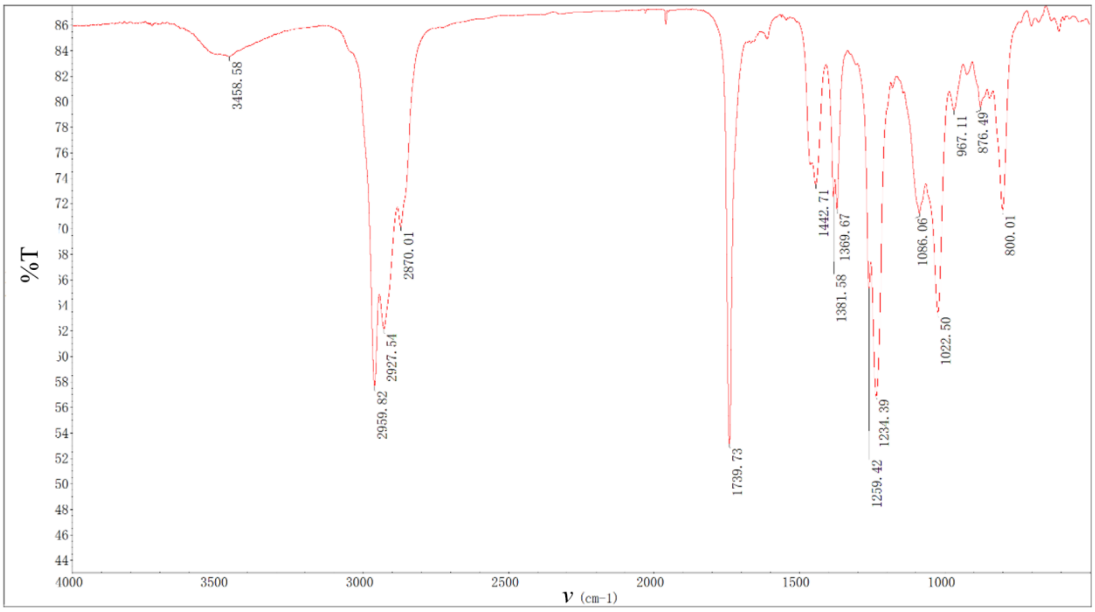

Figure S44. IR spectrum of **5**

EI202101071_A8-D5A2C3D4-4 -c1#9 RT: 1.86
T: + c EI Full ms [49.50-800.50]
m/z= 48-803

m/z	Intensity	Relative	Theo. Mass	Delta (mmu)	RDB equiv.	Composition
69.0694	3628504.0	57.83	69.0699	-0.48	1.5	$C_5 H_9$
93.0307	6274697.0	100.00	93.0335	-2.76	4.5	$C_6 H_5 O_1$
94.0432	957975.0	15.27	94.0413	1.86	4.0	$C_6 H_6 O_1$
106.0436	1595572.0	25.43	106.0413	2.25	5.0	$C_7 H_6 O_1$
115.0150	499380.0	7.96	115.0178	-2.83	7.5	$C_8 H_3 O_1$
119.0857	5229835.0	83.35	119.0855	0.18	4.5	$C_9 H_{11}$
120.0962	1191436.0	18.99	120.0934	2.84	4.0	$C_9 H_{12}$
131.0853	2099264.0	33.46	131.0855	-0.18	5.5	$C_{10} H_{11}$
132.0920	692026.0	11.03	132.0934	-1.35	5.0	$C_{10} H_{12}$
133.1009	3419703.0	54.50	133.1012	-0.27	4.5	$C_{10} H_{13}$
134.1082	1704048.0	27.16	134.1090	-0.84	4.0	$C_{10} H_{14}$
135.1163	2579477.0	41.11	135.1168	-0.49	3.5	$C_{10} H_{15}$
136.1240	1458241.0	23.24	136.1247	-0.65	3.0	$C_{10} H_{16}$
137.0960	395629.0	6.31	137.0961	-0.07	3.5	$C_9 H_{13} O_1$
137.1324	1104549.0	17.60	137.1325	-0.07	2.5	$C_{10} H_{17}$
143.0857	795009.0	12.67	143.0855	0.19	6.5	$C_{11} H_{11}$
145.1010	2868491.0	45.72	145.1012	-0.15	5.5	$C_{11} H_{13}$
146.1074	963911.0	15.36	146.1090	-1.65	5.0	$C_{11} H_{14}$
147.1164	3879065.0	61.82	147.1168	-0.38	4.5	$C_{11} H_{15}$
148.1232	1232813.0	19.65	148.1247	-1.44	4.0	$C_{11} H_{16}$
149.1321	1695247.0	27.02	149.1325	-0.35	3.5	$C_{11} H_{17}$
150.1400	5003048.0	79.73	150.1403	-0.33	3.0	$C_{11} H_{18}$
157.1015	877289.0	13.98	157.1012	0.29	6.5	$C_{12} H_{13}$
159.1144	2300180.0	36.66	159.1168	-2.47	5.5	$C_{12} H_{15}$
161.1300	1680451.0	26.78	161.1325	-2.47	4.5	$C_{12} H_{17}$
162.1380	1183344.0	18.86	162.1403	-2.29	4.0	$C_{12} H_{18}$
163.1457	1384555.0	22.07	163.1481	-2.47	3.5	$C_{12} H_{19}$
164.1553	585854.0	9.34	164.1560	-0.63	3.0	$C_{12} H_{20}$
171.1180	1045039.0	16.65	171.1168	1.15	6.5	$C_{13} H_{15}$
173.1321	1152068.0	18.36	173.1325	-0.38	5.5	$C_{13} H_{17}$
174.1385	433255.0	6.90	174.1403	-1.79	5.0	$C_{13} H_{18}$
175.1477	925399.0	14.75	175.1481	-0.43	4.5	$C_{13} H_{19}$
177.1279	384613.0	6.13	177.1274	0.52	4.5	$C_{12} H_{17} O_1$
185.1322	892351.0	14.22	185.1325	-0.28	6.5	$C_{14} H_{17}$
187.1477	4182551.0	66.66	187.1481	-0.43	5.5	$C_{14} H_{19}$
189.1278	385676.0	6.15	189.1274	0.40	5.5	$C_{13} H_{17} O_1$
189.1628	804874.0	12.83	189.1638	-1.01	4.5	$C_{14} H_{21}$
199.1476	434672.0	6.93	199.1481	-0.52	6.5	$C_{15} H_{19}$
201.1632	578854.0	9.23	201.1638	-0.61	5.5	$C_{15} H_{21}$
202.1705	967219.0	15.41	202.1716	-1.10	5.0	$C_{15} H_{22}$
203.1796	1654107.0	26.36	203.1794	0.14	4.5	$C_{15} H_{23}$
215.1782	714915.0	11.39	215.1794	-1.19	5.5	$C_{16} H_{23}$
217.1946	1083846.0	17.27	217.1951	-0.51	4.5	$C_{16} H_{25}$
227.1794	1617101.0	25.77	227.1794	-0.01	6.5	$C_{17} H_{23}$
230.2022	873007.0	13.91	230.2029	-0.68	5.0	$C_{17} H_{26}$

EI202101071_A8-D5A2C3D4-4 -c1#9 RT: 1.86
T: + c EI Full ms [49.50-800.50]
m/z= 48-803

m/z	Intensity	Relative	Theo. Mass	Delta (mmu)	RDB equiv.	Composition
245.1905	1828265.0	29.14	245.1900	0.49	5.5	$C_{17}H_{25}O_1$
255.2119	485174.0	7.73	255.2107	1.13	6.5	$C_{19}H_{27}$
270.2332	553869.0	8.83	270.2342	-1.05	6.0	$C_{20}H_{30}$
288.2451	1056557.0	16.84	288.2448	0.34	5.0	$C_{20}H_{32}O_1$

Figure S45. HREIMR spectrum of **5**

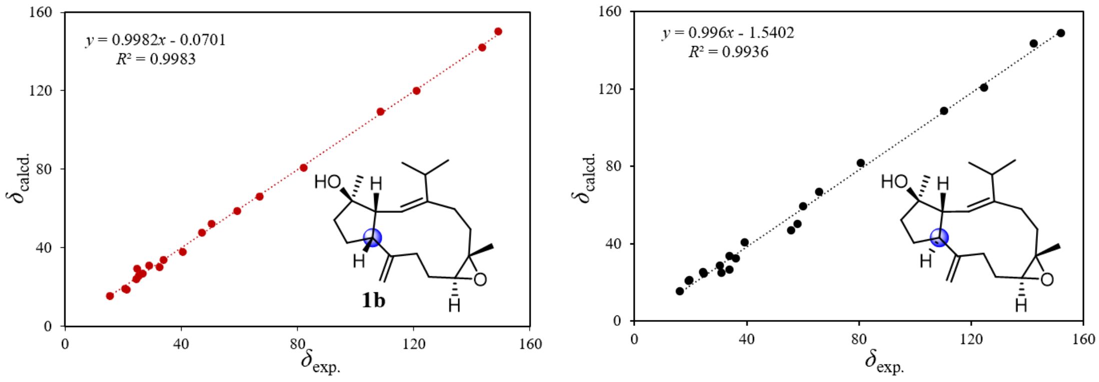

3R*, 4R*, 7R*, 11S*, 12S*-**1a**
DP4+ (all date): 100%

3R*, 4R*, 7S*, 11S*, 12S*-**1b**
DP4+ (all date): 0%

Figure S46. Regression analysis of experimental *vs* calculated ^{13}C NMR chemical shifts of (3R*,4R*,7R*,11S*,12S*)-**1a**, (3R*,4R*,7S*,11S*,12S*)-**1b** at the PCM/mPW1PW91/6-31+G** level using DP4+ method.

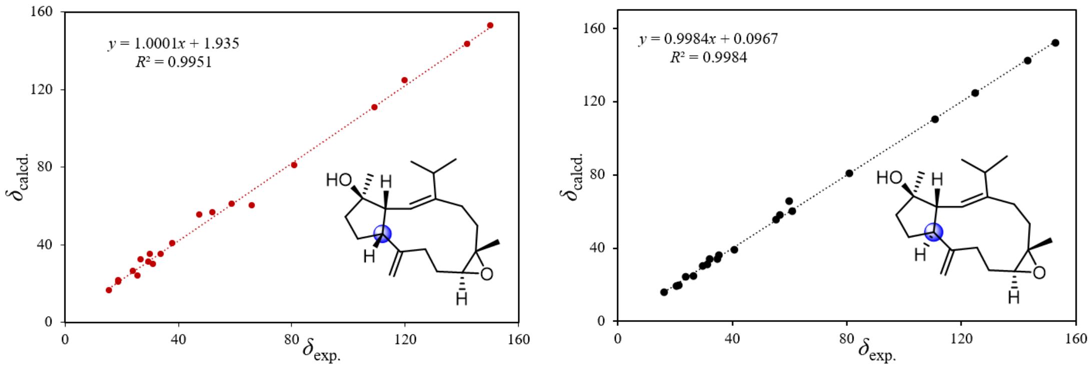

3R*, 4R*, 7R*, 11S*, 12S*-**2a**
DP4+ (all date): 0%

3R*, 4R*, 7S*, 11S*, 12S*-**2b**
DP4+ (all date): 100%

Figure S47. Regression analysis of experimental *vs* calculated ^{13}C NMR chemical shifts of (3R*,4R*,7R*,11S*,12S*)-**2a**, (3R*,4R*,7S*,11S*,12S*)-**2b** at the PCM/mPW1PW91/6-31+G** level using DP4+ method

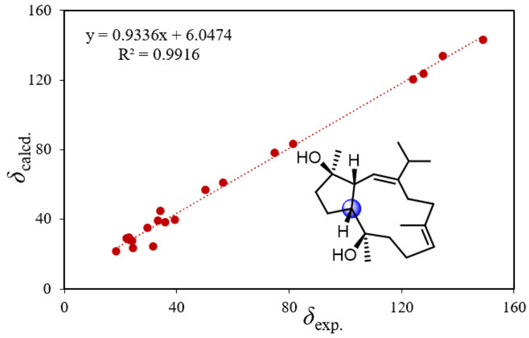

3R*, 4R*, 7R*, 8R*-**6a**

DP4+ (all date): 0.02%

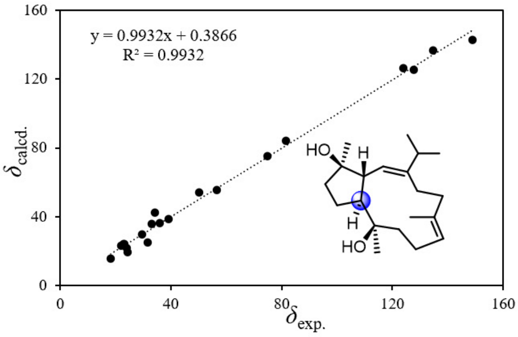

3R*, 4R*, 7S*, 8R*-**6b**

DP4+ (all date): 99.98%

Figure S48. Regression analysis of experimental *vs* calculated ^{13}C NMR chemical shifts of (3*R**,4*R**,7*R**,8*R**)-**6a**, (3*R**,4*R**,7*S**,8*R**)-**6b** at the PCM/mPW1PW91/6-31+G** level using DP4+ method.

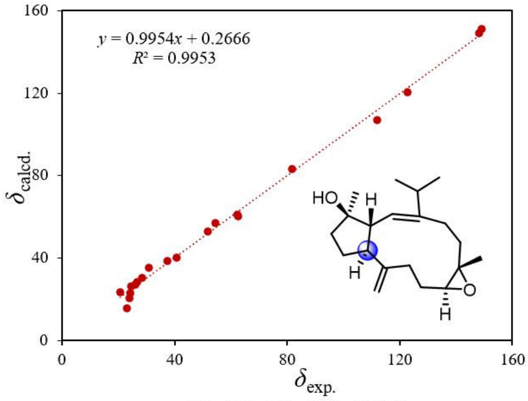

3R*, 4R*, 7S*, 11S*, 12S*-**7a**

DP4+ (all date): 100%

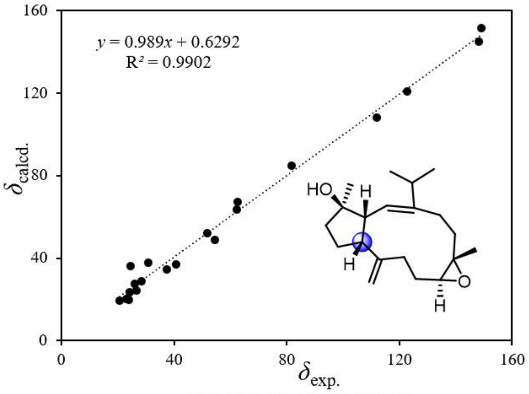

3R*, 4R*, 7R*, 11S*, 12S*-**7b**

DP4+ (all date): 0%

Figure S49. Regression analysis of experimental *vs* calculated ^{13}C NMR chemical shifts of (3*R**,4*R**,7*S**,11*S**,12*S**)-**7a**, (3*R**,4*R**,7*R**,11*S**,12*S**)-**7b** at the PCM/mPW1PW91/6-31+G** level using DP4+ method.

Table S1 Crystal data and structure refinement for compound **1**

Empirical formula	$C_{20}H_{32}O_2$
Formula weight	304.45
Temperature/K	170
Crystal system	monoclinic
Space group	$P2_1$
a/Å	6.3207(2)
b/Å	16.7821(6)
c/Å	8.6966(3)
α/°	90
β/°	101.226(2)
γ/°	90
Volume/$Å^3$	904.84(5)
Z	2
$\rho_{calc}g/cm^3$	1.117
μ/mm^{-1}	0.536
F(000)	336.0
Crystal size/mm^3	0.15 × 0.08 × 0.05
Radiation	CuKα (λ = 1.54178)
2Θ range for data collection/°	10.37 to 149.108
Index ranges	$-7 \le h \le 7$, $-20 \le k \le 20$, $-10 \le l \le 10$
Reflections collected	21562
Independent reflections	3673 [R_{int} = 0.0440, R_{sigma} = 0.0272]
Data/restraints/parameters	3673/1/204
Goodness-of-fit on F^2	1.047
Final R indexes [I>=2σ (I)]	R_1 = 0.0313, wR_2 = 0.0796
Final R indexes [all data]	R_1 = 0.0324, wR_2 = 0.0805
Largest diff. peak/hole / e $Å^{-3}$	0.14/-0.13
Flack parameter	-0.06(7)
Final R indexes [I>=2σ (I)]	R_1 = 0.0313, wR_2 = 0.0796
Final R indexes [all data]	R_1 = 0.0324, wR_2 = 0.0805
Largest diff. peak/hole / e $Å^{-3}$	0.14/-0.13
